# Asarinin attenuates bleomycin-induced pulmonary fibrosis by activating PPARγ

**DOI:** 10.1038/s41598-023-41933-5

**Published:** 2023-09-07

**Authors:** Qian Zeng, Ting-ting Zhou, Wen-jie Huang, Xiao-ting Huang, Lei Huang, Xiao-hua Zhang, Xiao-xue Sang, Yu-yang Luo, Yu-mei Tian, Bin Wu, Lin Liu, Zi-qiang Luo, Bin He, Wei Liu, Si-yuan Tang

**Affiliations:** 1 Xiangya Nursing School, Central South University, 172 Tongzipo Road, Changsha, 410013 Hunan China; 2 School of Nursing, Hunan University of Medicine, Huaihua, Hunan China; 3 Hunan Prevention and Treatment Institute for Occupational Diseases, Changsha, China; 4 Xiangya School of Medicine, Central South University, Changsha, Hunan China

**Keywords:** Chronic obstructive pulmonary disease, Pharmacodynamics

## Abstract

Idiopathic pulmonary fibrosis (IPF) is a chronic progressive interstitial lung disease that lacks effective treatment modalities. Once patients are diagnosed with IPF, their median survival is approximately 3–5 years. PPARγ is an important target for the prevention and treatment of pulmonary fibrosis. Asarinin is a lignan compound that can be extracted from food plant *Asarum heterotropoides*. In this study, we investigated the therapeutic effects of asarinin in a pulmonary fibrosis model constructed using bleomycin in mice and explored the underlying mechanisms. Intraperitoneal administration of asarinin to mice with pulmonary fibrosis showed that asarinin effectively attenuated pulmonary fibrosis, and this effect was significantly inhibited by the PPARγ inhibitor GW9662. Asarinin inhibited TGF-β1-induced fibroblast-to-myofibroblast transition *in vitro*, while GW9662 and PPARγ gene silencing significantly inhibited this effect. In addition, asarinin inhibited not only the canonical Smad pathway of TGF-β but also the non-canonical AKT and MAPK pathways by activating PPARγ. Our study demonstrates that asarinin can be used as a therapeutic agent for pulmonary fibrosis, and that PPARγ is its key target.

## Introduction

Idiopathic pulmonary fibrosis (IPF) is a chronic, progressive, and fibrotic lung disease characterized by abnormal accumulation of fibrous tissue in the lung parenchyma, replacement of healthy tissue by altered extracellular matrix, and disruption of alveolar structure^1^. These factors lead to decreased lung compliance, disruption of gas exchange, and ultimately, respiratory failure and death^2^. There is a lack of effective drug therapies for IPF, and the median survival of patients diagnosed with IPF is approximately 3–5 years^3^. Therefore, investigating effective pharmacological treatments for IPF is crucial.

Although the pathogenesis of IPF is not fully understood, interactions and transformations between multiple cell types, including myofibroblasts, play an important role in its development^4^. Myofibroblasts play a vital role in promoting extracellular matrix (ECM) deposition, inflammatory mediator release, and epithelial injury, all of which are thought to be key factors in perpetuating injury and promoting the fibrotic cycle^5,6^. Sustained activation of the cytokine transforming growth factor-β (TGF-β) plays an important role in maintaining the myofibroblast phenotype^7^. TGF-β is an indispensable mediator of fibrosis development and progression. Its upregulation has been demonstrated in a mouse model of experimental pulmonary fibrosis and in the bronchoalveolar lavage fluid from patients with IPF^8^. In normal physiology, TGF-β has multiple functions including immunomodulation, regulation of cell proliferation and differentiation, and apoptosis^9^. A large body of evidence suggests that TGF-β and its downstream signaling pathways, such as the Smad, MAPK, and AKT pathways, play a central role in the IPF pathogenesis^10–12^. In addition to the Smad pathway, the AKT and MAPK pathways can directly contribute to bleomycin (BLM)-induced lung fibrosis by regulating fibroblast viability, differentiation, and migration^13,14^. In summary, the focus of current research is studying the transition of fibroblasts to myofibroblasts under TGF-β induction and developing therapeutic tools targeting myofibroblasts.

Peroxisome proliferator-activated receptor γ (PPARγ) is a ligand-activated transcription factor belonging to the peroxisome proliferator-activated receptor superfamily^15^. PPARγ regulates many physiological activities, including adipocyte differentiation, glucose homeostasis, inflammation, immune responses, and proliferation^16^. Several studies have demonstrated the importance of PPARγ in preventing the development of pulmonary fibrosis. *In vitro*, activation of cellular PPARγ receptors using synthetic or natural PPARγ ligands can block *in vitro*-induced profibrotic responses, such as treatment of lung fibroblasts with PPARγ agonists, which inhibit TGF-β-induced collagen and fibronectin synthesis, myofibroblast differentiation, fibroblast migration, and secretion of fibrogenic growth factors such as TGF-β^17–20^. *In vivo*, various PPARγ agonists, including troglitazone and rosiglitazone, were effective in inhibiting BLM-induced pulmonary fibrosis, and PPARγ agonists were also effective in inhibiting paraquat-induced pulmonary fibrosis^21–25^. In addition, PPARγ also plays an important role in fibrosis of other organs, such as liver fibrosis and systemic sclerosis^26–28^. Overall, the activation of PPARγ negatively regulates the fibrosis process, suggesting that this receptor is a key target for pulmonary fibrosis treatment.

The lignan compound asarinin, which can be extracted from *Asarum heterotropoides*^29,30^, has been shown to have a wide range of pharmacological effects, including anti-inflammatory^31^, anti-immune rejection^32^, anti-allergic^33^, and anti-cancer effects^34^, but its specific role in pulmonary fibrosis-related diseases remains unclear. In this study, for the first time, we observed the anti-pulmonary fibrosis effect of asarinin in a BLM-induced mouse model of pulmonary fibrosis and explored its mechanism of action by inhibiting myofibroblast transformation.

## Materials and methods

Any supporting data within the article are available from the corresponding author upon reasonable request.

### Experimental animals and pulmonary fibrosis model

Eight-week-old male C57BL/6 mice weighing 20–22 g and SD rats, were ordered from Central South University. They lacked underlying diseases and were housed in a pathogen-specific barrier environment at the Department of Zoology, Central South University. Mice were anesthetized with sodium pentobarbital after one week of adaptive feeding, and 50 μL of BLM (3 mg/kg) (Nippon Kayaku, Tokyo, Japan) or an equivalent amount of saline was injected intratracheally. Saline containing 5% dimethyl sulfoxide (Macklin, Shanghai, China) and 40% polyethylene glycol 300 (PEG300) (Macklin) with 5% Tween80 (Macklin) (v/v/v) was used as the solvent to dilute asarinin (Solarbio, Beijing, China). Animal experiments included therapeutic and inhibitor projects.

#### Therapeutic study

Of 110 mice, 10 were randomly selected as the control group, and the rest were used for BLM modeling. In the control group, mice were injected intraperitoneally with the drug solvent on d 15–28 after intratracheal injection of saline, and on d 14 after intratracheal injection of BLM, mice were stratified and sampled according to body weight into four groups with the same number of mice: pulmonary fibrosis model, injected intraperitoneally with the drug solvent on d 15–28; high-dose, injected intraperitoneally with 20 mg/kg of asarinin on d 15–28; medium-dose, injected intraperitoneally with 5 mg/kg of asarinin on d 15–28; low-dose group, injected intraperitoneally with 1 mg/kg of asarinin on d 15–28. The mice were euthanized on d 29 by exsanguination of the femoral artery after anesthesia with sodium pentobarbital and the lung tissue was removed.

#### Inhibitor study

Ten mice were randomly selected from 100 mice as controls, and the rest were used for modeling. The groups were: control group—intraperitoneal injection of solvent on d 15–28 after intratracheal injection of saline; pulmonary fibrosis model—mice were stratified and sampled by body weight on d 14 after intratracheal injection of BLM. They were then divided into three groups with the same number of mice: a pulmonary fibrosis group receiving intraperitoneal injection of solvent on d 15–28, a high-dose group receiving intraperitoneal injection of 20 mg/kg of asarinin on d 15–28, and an inhibitor group receiving intraperitoneal injection of 1 mg/kg GW9662 (Sigma, St. Louis, MO, USA) for 30 min, followed by intraperitoneal injection of 20 mg/kg asarinin on d 15–28.

### Hematoxylin and eosin staining, Masson staining, and Ashcroft score

Mouse lung tissues were fixed with 4% paraformaldehyde (Servicebio, Wuhan, China), embedded in paraffin, and sectioned. Sections were stained with hematoxylin and eosin (Servicebio) and Masson’s dye solution (Servicebio). The Ashcroft score refers to the methodology of Ashcroft et al., which is the average of the fibrosis scores of hematoxylin and eosin (HE)-stained sections assessed by two researchers for each animal^35^.

### Immunohistochemistry

The paraffin sections were successively deparaffinized with xylene and dehydrated with ethanol. After repairing the antigen with microwave, membranes were broken with Triton X-100 (Servicebio), then incubated with goat serum after blocking endogenous peroxidase. They were subsequently incubated with monoclonal antibodies to smooth muscle actin (Proteintech, Wuhan, China) or collagen type I (Proteintech) overnight at 4 ℃. The samples were incubated with a goat anti-rabbit IgG monoclonal antibody (Proteintech) for 1 h at room temperature. The nuclei were stained with hematoxylin after staining with DAB chromogenic solution.

### Hydroxyproline determination

According to the instructions of the hydroxyproline assay kit (Nanjing Jiancheng Biotechnology Institute, Nanjing, China), mouse lung tissue was homogenized, and each reagent in the kit was added in turn, followed by centrifugation at 3500 rpm for 10 min. The supernatant was transferred to a 96-well plate, the absorbance value was measured at 550 nm, and the hydroxyproline content was calculated according to the absorbance value.

### RNA extraction and quantitative real-time PCR

Total RNA was extracted from lung tissues and cells using TRIzol reagent (Thermo Fisher Scientific, Waltham, MA, USA). RNA was reverse-transcribed into cDNA using a reverse transcription kit (Thermo Fisher Scientific). Quantitative real-time PCR (Q-PCR) was performed. The Q-PCR conditions were: 95 °C for 2 min, followed by 40 cycles of 95 °C for 3 s and 60 °C for 30 s, with a melting curve of 60–95 °C. The primer sequences used in this experiment are listed in Table 1 (Sangon Biotech).

**Table 1:** The primer sequences.

Gene	Forward [5'-3']	Reverse [5'-3']
Mouse β-Actin	GTGCTATGTTGCTCTAGACTTCG	ATGCCACAGGATTCCATACC
Mouse α-SMA	TGGCTATTCAGGCTGTGCTGTC	CAATCTCACGCTCGGCAGTAGT
Mouse collagen I	GAGCGGAGAGTACTGGATCG	GCTTCTTTTCCTTGGGGTTC
Mouse PPARɣ	AGCCCTTTACCACAGTTGATTTCTCC	GCAGGTTCTACTTTGATCGCACTTTG
Rat β-Actin	TGTCACCAACTGGGACGATA	GGGGTGTTGAAGGTCTCAAA
Rat α-SMA	GCGTGGCTATTCCTTCGTGACTAC	CATCAGGCAGTTCGTAGCTCTTCTC
Rat collagen I	TGTTGGTCCTGCTGGCAAGAATG	GTCACCTTGTTCGCCTGTCTCAC
Rat PPARɣ	CGCCAAGGTGCTCCAGAAGATG	AGGGTGAAGGCTCATATCTGTCTCC
Human β-Actin	CCTGGCACCCAGCACAAT	GGGCCGGACTCGTCATAC
Human α-SMA	TCCGGAGCGAAATACTCTG	CCCGGCTTCATCGTATTCCT
Human collagen I	CCACCAATCACCTGCGTACA	CACGTCATCGCACAACACCT
Human PPARɣ	TGAATCCAGAGTCCGCTGACCTC	ATCGCCCTCGCCTTTGCTTTG

### Western blot

Total protein was extracted from mouse lung tissue or cells using RIPA lysis buffer containing phenylmethylsulfonyl fluoride (PMSF), phosphatase inhibitors, and protease inhibitors. Proteins were subjected to 10% SDS-PAGE after the total protein concentration was determined with the BCA kit (Cwbio, Jiangsu, China), and then transferred to PVDF membranes and blocked with 5% skim milk. Subsequently, the membrane was incubated with antibodies (all antibodies ordered from Proteintech except that P-Smad3 ordered from AiFang biological, Changshan, China) against β-actin, α-SMA, Collagen I, PPARγ, Smad3, P-smad3, AKT, P-AKT, p38, P-p38, ERK, P-ERK, JNK, and P-JNK at 4 ℃ overnight. After incubation with a horseradish peroxidase-labeled goat anti-rabbit or horseradish peroxidase-labeled goat anti-mouse IgG monoclonal antibodies for 2 h at room temperature, protein expression was observed using an ECL developer after TBST washing (Cwbio). Membranes were cut horizontally, and individual gels of close/overlapping membrane strips in MW were run for separate proteins, and loading controls for all gels were showed in Supplementary Material.

### Cell extraction and cell culture

After euthanasia, the chest cavity was exposed, the lung tissue was washed with PBS containing 1% penicillin–streptomycin solution (Procell, Wuhan, China) mixture, and the lung tissue was cut into pieces and placed in 1 mg/mL collagenase type I (Gibco, Waltham, MA, USA), followed by a water bath at 37 °C for 1 h. After centrifugation, the lung tissue was resuspended and filtered through a 70 µm filter, and the filtered liquid was collected. The cells were lysed with red blood cell lysate (Solarbio) for 5 min, resuspended, filtered through a 40 µm filter, and the filtrate was collected. After centrifugation at 1500 *rpm* for 10 min, the cells were resuspended in high-glucose Dulbecco’s modified Eagle’s medium (Procell) containing 20% FBS (BI, Beit Haemek, Israel) and 1% penicillin–streptomycin solution. Passages 3–7 of primary lung fibroblasts were used in the experiment. Human lung fibroblasts (HFL-1) (Procell) were cultured in Ham’s F-12K medium (Procell) containing 10% FBS and 1% penicillin–streptomycin solution. All cells were cultured at 37 °C in a humidified incubator with 5% carbon dioxide.

### Cell viability assay

Cell viability was measured using the Cell Counting Kit-8 (CCK-8) (Elabscience, Wuhan, China). Cells were seeded in a 96-well plate and incubated with different concentrations of asarinin for 24 h. After washing the cells with PBS, CCK-8 reagent was added and the cells were incubated at 37 °C for 30 min. The absorbance of each well was measured at a wavelength of 450 nm.

### Immunofluorescence

After the intervention, the cells were first fixed with 4% paraformaldehyde (Servicebio) for 30 min, the membrane was broken with 0.4% Triton X (Abiowell, Changsha, China) for 10 min, and then incubated with endogenous peroxidase blocking agent (Beyotime Biotechnology, Shanghai, China) for 10 min. After blocking with goat serum for 30 min, the PPARγ antibody and Smad3 antibody (Proteintech) were incubated overnight. After rewarming at room temperature for 30 min, the antibody was incubated with a fluorescent secondary antibody (Abiowell) for 1 h and then observed using a confocal microscope.

### siRNA transfection

After the cell density reached 60–80%, siRNA reagent (Santa Cruz Biotechnology, TX, USA) per 3 µL and Lipofectamine 2000 (Invitrogen, Waltham, MA, USA) per 7 µL were diluted and mixed with 100 µL DMEM, respectively, and allowed to stand at room temperature for 30 min. After washing the cells twice with DMEM, 800 µL of siRNA and Lipofectamine 2000 mixture were added to each well, and the cells were incubated for 7 h. Then, 1 mL of medium containing double serum and penicillin–streptomycin solution was added, the mixture was incubated for 24 h and then replaced with conventional medium.

### Statistical analysis

All data were analyzed using GraphPad Prism 8.3.1. All data are expressed as mean ± standard deviation, and the t-test was used to compare the measurement data between the two groups. One-way analysis of variance (ANOVA) was used to compare the measurement data of multiple groups. Tukey’s test was used to compare multiple groups of measured data. P < 0.05 was considered statistically significant.

### Ethics declarations

The subjects of this study were mice and fibroblasts, where mouse and rat-derived fibroblasts were obtained from mice and rats ordered from Central South University and Human fetal lung fibroblast (HFL-1) was the ordered cell line. No tissues were taken directly from patients for this study. This study was carried out in accordance with the he welfare and ethical principles of laboratory animal and this study was carried out in compliance with ARRIVE guidelines. No human subjects were involved in this study and the experimental protocol was approved by the Laboratory Animal Welfare and Ethical Committee of Central South University. (IACUC number: CSU-2022-0051).

## Results

### Asarinin attenuated BLM-induced pulmonary fibrosis in mice

**Figure 1:**
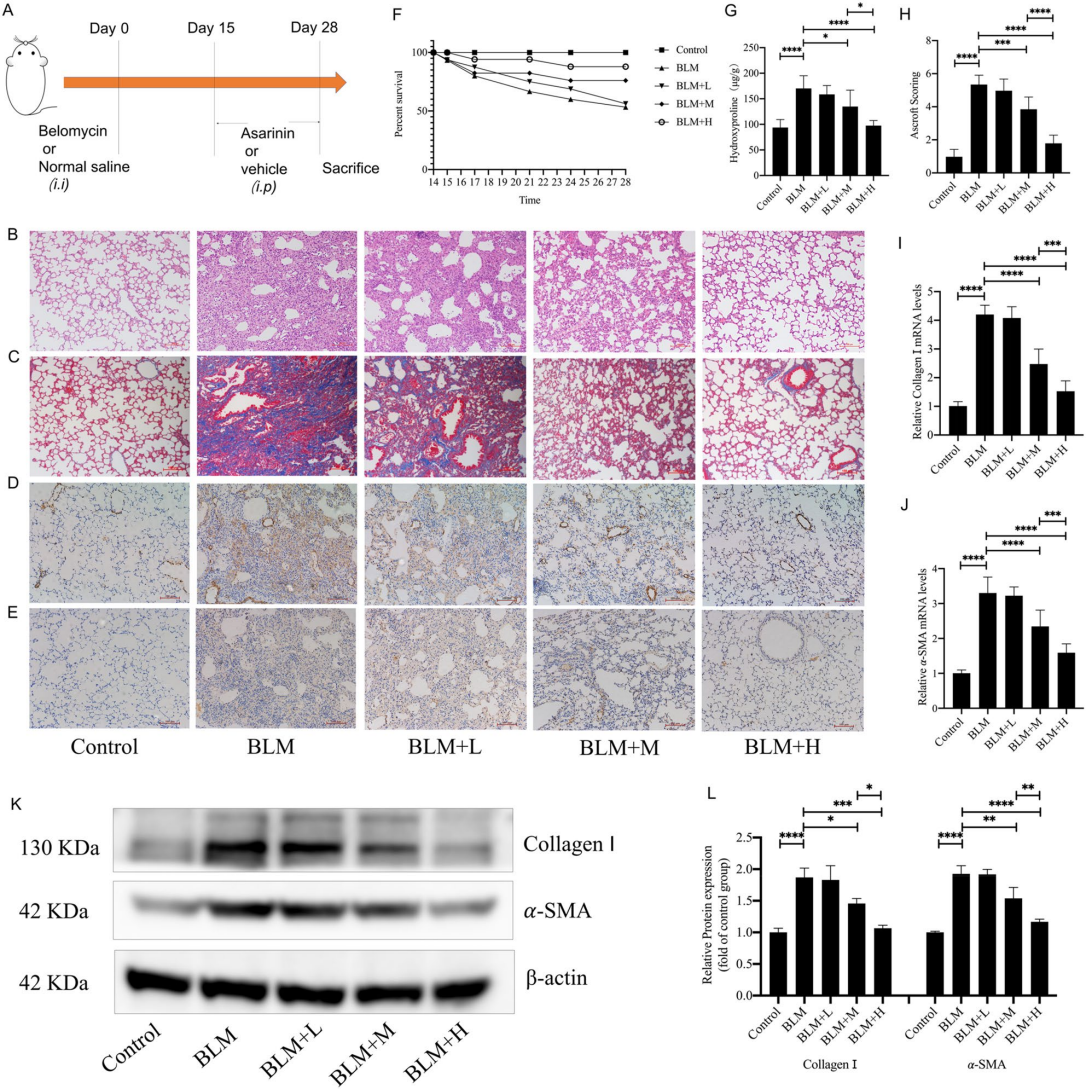
Asarinin improved lung tissue morphology and structure and reduced lung fibrosis marker levels. A mouse lung fibrosis model was constructed using BLM, and high (20 mg/kg), medium (5 mg/kg), and low (1 mg/kg) doses of asarinin were injected intraperitoneally to assess the direct antifibrotic effect of asarinin (**A**). Hematoxylin and eosin staining and Masson staining analysis for changes in lung tissue structure and extracellular matrix deposition (magnification ×100) (**B,C**). Ashcroft score analysis for the degree of pulmonary fibrosis (**H**). Record the number of deaths in each group of mice and make a survival curve (**F**). Immunohistochemical analysis for the expression of α-SMA and type I collagen in lung tissue (magnification ×100) (**D**,**E**). Biochemical methods were used to determine the hydroxyproline content of lung tissue (**G**). Quantitative real-time PCR analysis for the gene expression levels of *Acta2* with *Col1a1* in lung tissue (**I**,**J**). Western blot analysis of α-SMA and type I collagen protein levels in lung tissue (**K**,**L**). Control represents the control group; BLM represents the pulmonary fibrosis model group; BLM+L represents the low-dose asarinin treatment group; BLM +M represents the medium-dose asarinin treatment group; BLM + H represents the high-dose asarinin treatment group. Data are expressed as mean ± standard deviation, sample size (n) = 8 for each group, *P < 0.05; **P < 0.01; ***P < 0.001; ****P < 0.0001.

Since fibrosis in mice became progressively more severe from d 15–28 after intratracheal injection of BLM^36,37^, we injected asarinin intraperitoneally to observe its direct antifibrotic effect (Fig. 1A). HE and Masson staining revealed that medium and high doses of asarinin effectively improved the accumulation of ECM and disorganized alveolar structure in the lung tissue of mice with pulmonary fibrosis (Fig. 1B,C), which was consistent with the Ashcroft score (Fig. 1H). Asarinin also improved survival in mice with pulmonary fibrosis (Fig. 1F). Medium and high doses of asarinin similarly reduced hydroxyproline content in the lung tissue of mice with pulmonary fibrosis (Fig. 1G). Immunohistochemical results showed that the expression levels of α-SMA and type I collagen in the lung tissues of mice with pulmonary fibrosis were reduced by medium and high doses of asarinin (Fig. 1D,E), which was consistent with the western blotting and quantitative real-time PCR results (Fig. 1I–L). The above results show that a high dose of asarinin had more obvious effects than a medium dose. In conclusion, asarinin effectively attenuates BLM-induced pulmonary fibrosis in mice.

### Asarinin attenuated the reduction of PPARγ and activation of Smad, AKT, and MAPK in BLM-induced pulmonary fibrosis in mice

**Figure 2:**
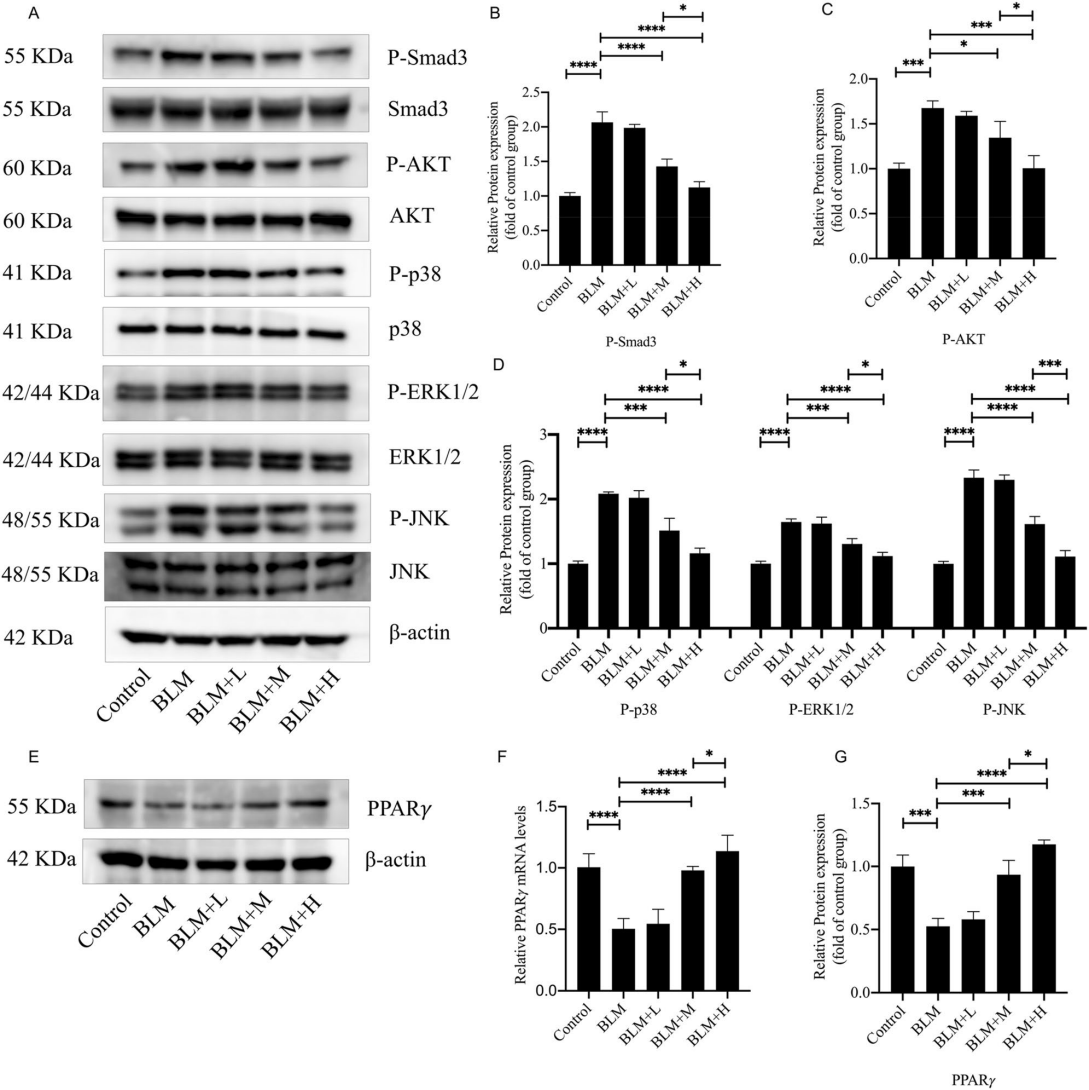
Asarinin attenuated the decrease in expression of PPARγ and increase in phosphorylation levels of Smad3, AKT, p38, ERK1/2 and JNK caused by BLM. Western blotting analysis for protein expression levels of P-Smad3, Smad3, P-AKT, AKT, p38, P-p38, ERK1/2, P-ERK1/2, JNK, P-JNK and PPARγ protein expression levels in mouse lung tissues (**A–E,G**), and quantitative real-time PCR analysis for gene expression levels of *Pparg* in mouse lung tissues (**F**). Data are expressed as mean ± standard deviation, sample size (n) = 8 for each group, *P < 0.05; **P < 0.01; ***P < 0.001; ****P < 0.0001.

While we observed that asarinin attenuated BLM-induced pulmonary fibrosis, we also found that the phosphorylation levels of Smad3, AKT, p38, ERK1/2, and JNK in the lung tissue of BLM-induced pulmonary fibrosis mice were significantly increased compared with those in the control group, and asarinin effectively reduced the phosphorylation levels of these proteins (Fig. 2A–D). In contrast, the protein and gene expression levels of PPARγ were significantly reduced in the lung tissue of BLM-induced pulmonary fibrosis mice compared with those in the control group and asarinin effectively inhibited this reduction (Fig. 2E–G). These results suggest that the attenuation of BLM-induced pulmonary fibrosis by asarinin may be related to its effects on PPARγ, Smad, AKT, and MAPK pathways.

### Asarinin inhibited TGF-β1-induced fibroblast-to-myofibroblast transition

**Figure 3:**
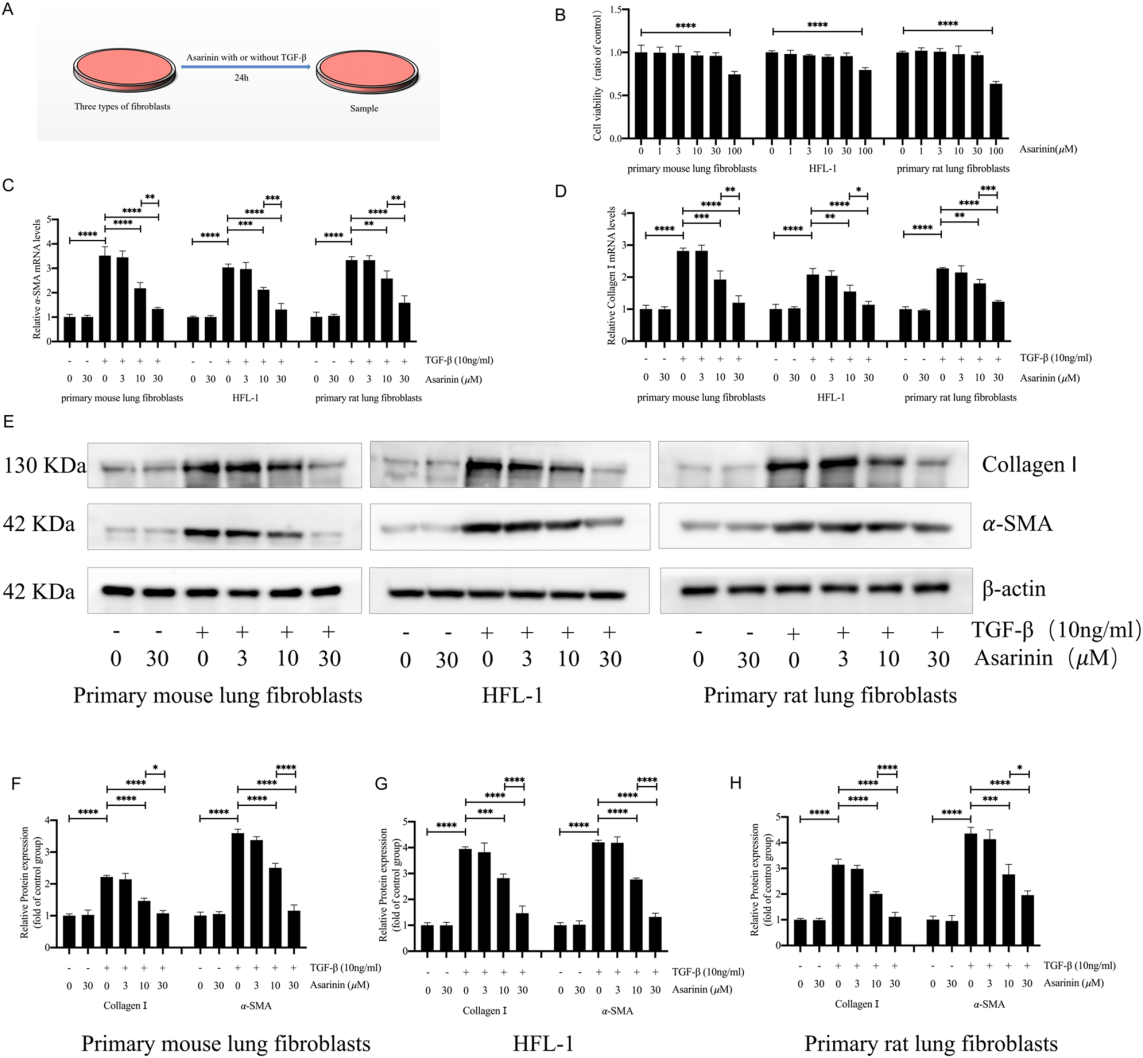
Asarinin decreased the expression levels of TGF-β1-induced α-SMA and type I collagen (**A**). CCK-8 analysis of the effects of different concentrations of asarinin on the cell viability of primary mouse lung fibroblasts, HFL-1 cells and primary rat lung fibroblasts were examined (**B**). Quantitative real-time PCR analysis for the gene expression levels of *Acta2* with *Col1a1* in three types of cells induced by TGF-β1 (**C**,**D**). Western blotting analysis for the protein expression levels of α-SMA and type I collagen in the three types of cells induced by TGF-β1 (**E–H**). Data are expressed as mean ± standard deviation, and all experiments were repeated independently at least 3 times, sample size (n) = 3 for each group, *P < 0.05; **P < 0.01; ***P < 0.001; ****P < 0.0001.

We further explored the antifibrotic mechanism of asarinin by exploring the inhibitory effect of asarinin on TGF-β1 (10 ng/mL)-induced fibroblast-to-myofibroblast transition (Fig. 3A). We selected primary mouse lung fibroblasts, HFL-1 cells, and primary rat lung fibroblasts for this part of the experiment. First, we explored the effect of several concentrations of asarinin (1, 3, 10, 30, and 100 μM) on the viability of the three cell types. The results showed that 100 μM of asarinin significantly affected the viability of all cells (Fig. 3B). Therefore, we chose 30, 10, and 3 μM for subsequent experiments. Co-incubation of the cells with asarinin and TGF-β1 for 24 h showed that 10 or 30 μM of asarinin significantly reduced the protein and gene expression levels of the TGF-β1-induced myofibroblast markers α-SMA and type I collagen (Fig. 3C–H), and that the effect of 30 μM was more pronounced than that of 10 μM. This indicated that asarinin could effectively inhibit the transformation of fibroblasts into myofibroblasts *in vitro*.

### Asarinin promoted PPARγ expression and activated PPARγ

**Figure 4:**
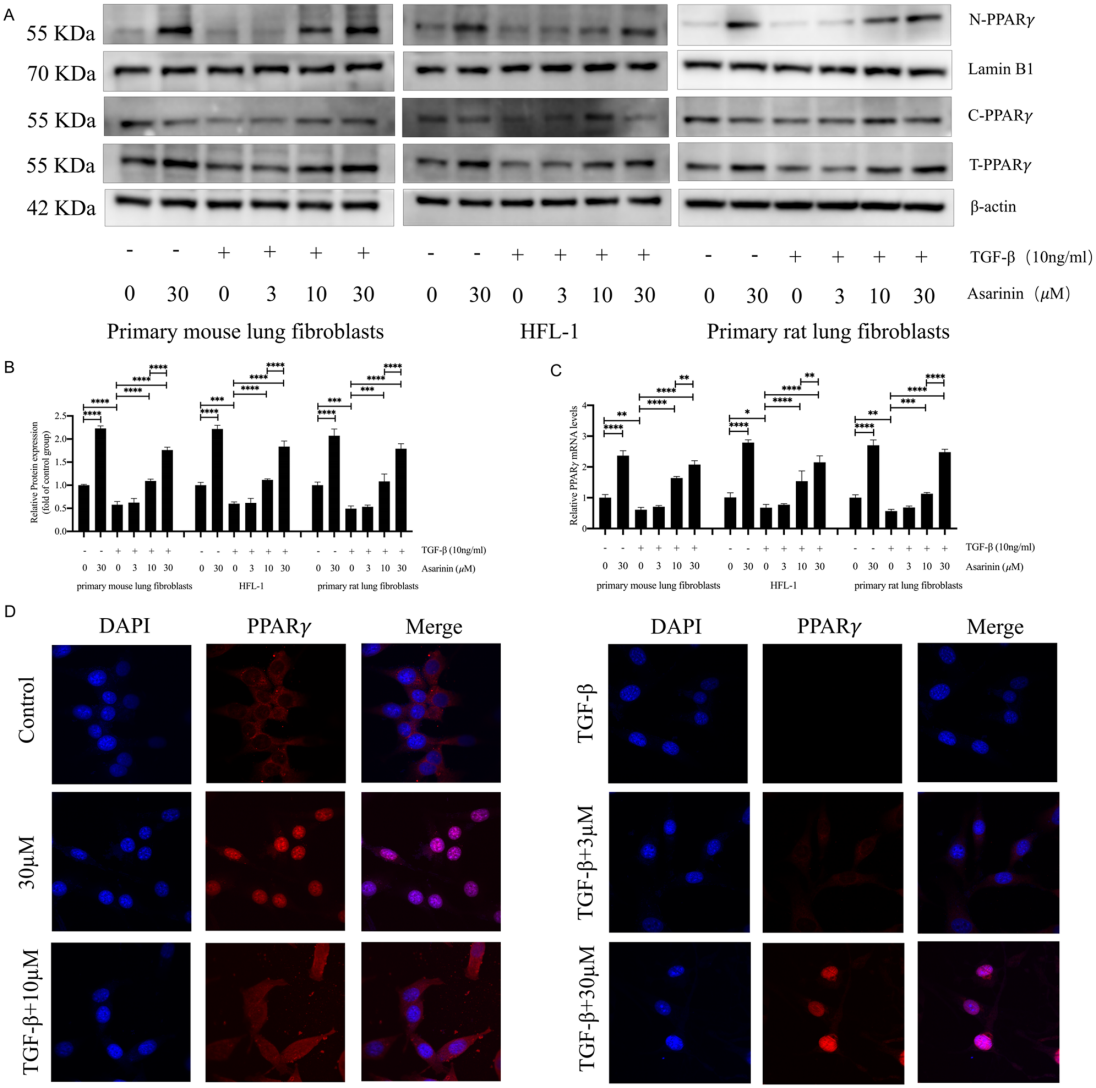
Asarinin promoted the expression and nuclear translocation of PPARγ. After extracting total cellular, nuclear, and cytoplasmic proteins, we verified their PPARγ protein expression levels by western blotting (**A**,**B**). The RNA expression levels of *Pparg* were detected by quantitative real-time PCR (**C**). The expression and nuclear translocation of PPARγ in mouse primary lung fibroblasts was detected by immunofluorescence (magnification ×1000) (**D**). Data are expressed as mean ± standard deviation, and all experiments were repeated independently at least 3 times, sample size (n) = 3 for each group, *P < 0.05; **P < 0.01; ***P < 0.001; ****P < 0.0001.

*In vivo* experiments revealed that asarinin promoted PPARγ expression, while attenuating bleomycin-induced pulmonary fibrosis. To explore whether the inhibition of myofibroblast transition by asarinin was related to PPARγ, we investigated its translocation and expression. We found that in primary mouse lung fibroblasts, HFL-1, and primary rat lung fibroblasts, asarinin effectively promoted the gene and protein expression levels of PPARγ, and significantly promoted its nuclear translocation (Fig. 4A–C). We also verified by immunofluorescence that asarinin promoted the expression and nuclear translocation of PPARγ in primary mouse lung fibroblasts (Fig. 4D). The above results suggest that asarinin may exert its antifibrotic effects through activation of PPARγ.

### PPARγ mediates the antifibrotic effect of asarinin

**Figure 5:**
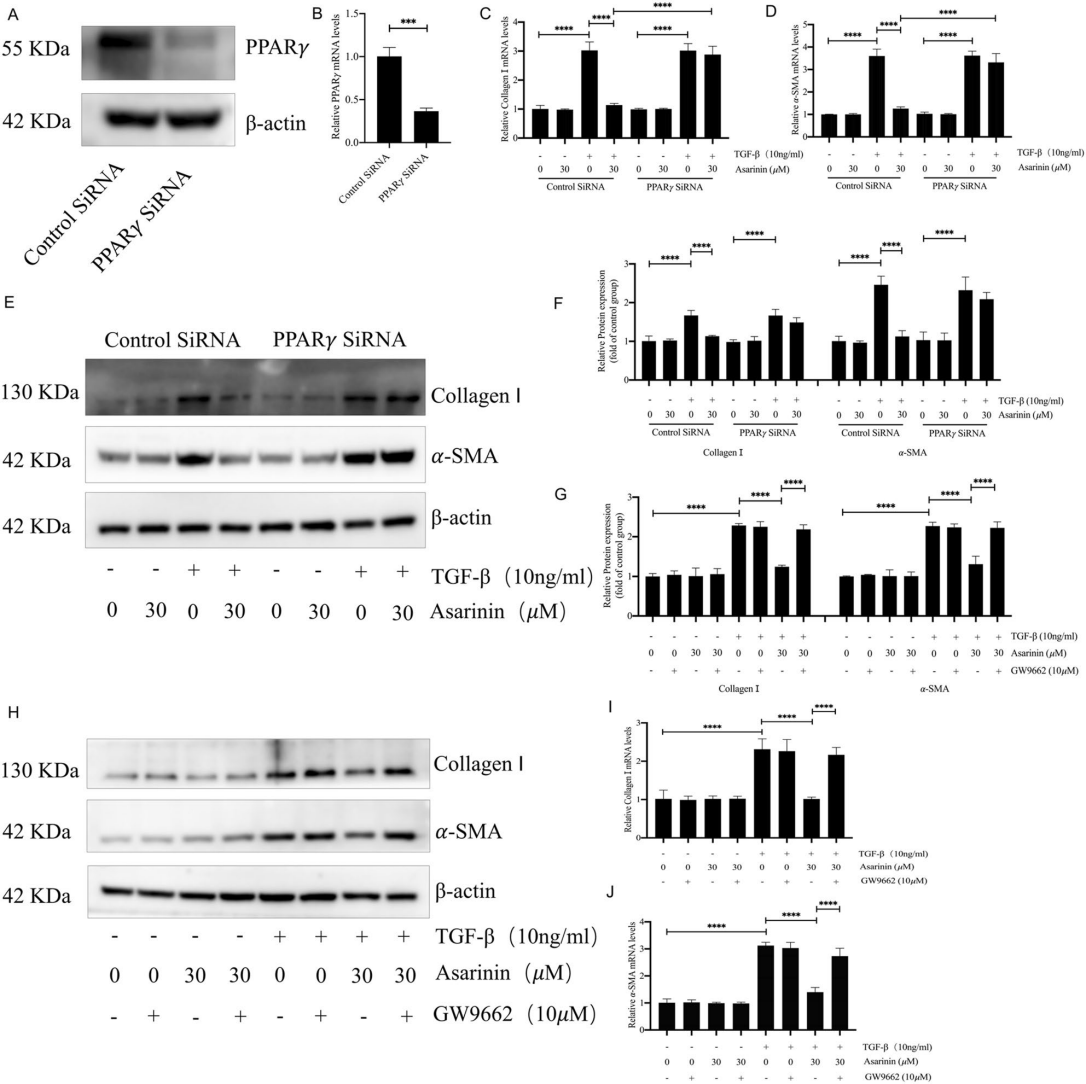
GW9662 and *Pparg* silencing inhibited the effect of asarinin on α-SMA and type I collagen expression in myofibroblast transition. Western blotting (WB) and quantitative real-time PCR (Q-PCR) verified the effect of *Pparg* silencing (**A**,**B**). WB detected the protein expression levels of α-SMA and type I collagen after *Pparg* silencing or GW9662 co-incubation (**E–H**). Q-PCR was performed to detect the gene expression levels of *Acta2* with *Col1a1* after *Pparg* silencing or GW9662 co-incubation (**C**,**D**,**I**,**J**). Data are expressed as mean ± standard deviation, and all experiments were repeated independently at least 3 times, sample size (n) = 3 for each group, *P < 0.05; **P < 0.01; ***P < 0.001; ****P < 0.0001.

In combination with our above results, to further determine the inhibitory effect of asarinin mediated by PPARγ on myofibroblast transition, we selected primary mouse lung fibroblasts and transfected siRNA targeting *Pparg* or used the *Pparg* inhibitor GW9662 to observe its role in the inhibition of myofibroblast transition by asarinin. Transfection with siRNA targeting *Pparg* inhibited the reduction in α-SMA and type I collagen expression after TGF-β1 induction (Fig. 5A–F). Similarly, the reduction in α-SMA and type I collagen expression was inhibited after GW9662 treatment (Fig. 5G–J). These results suggest that asarinin can inhibit TGF-β1-induced transition of fibroblasts to myofibroblasts by activating PPARγ.

### Asarinin inhibited the Smad pathway of TGF-β by activating PPARγ

**Figure 6:**
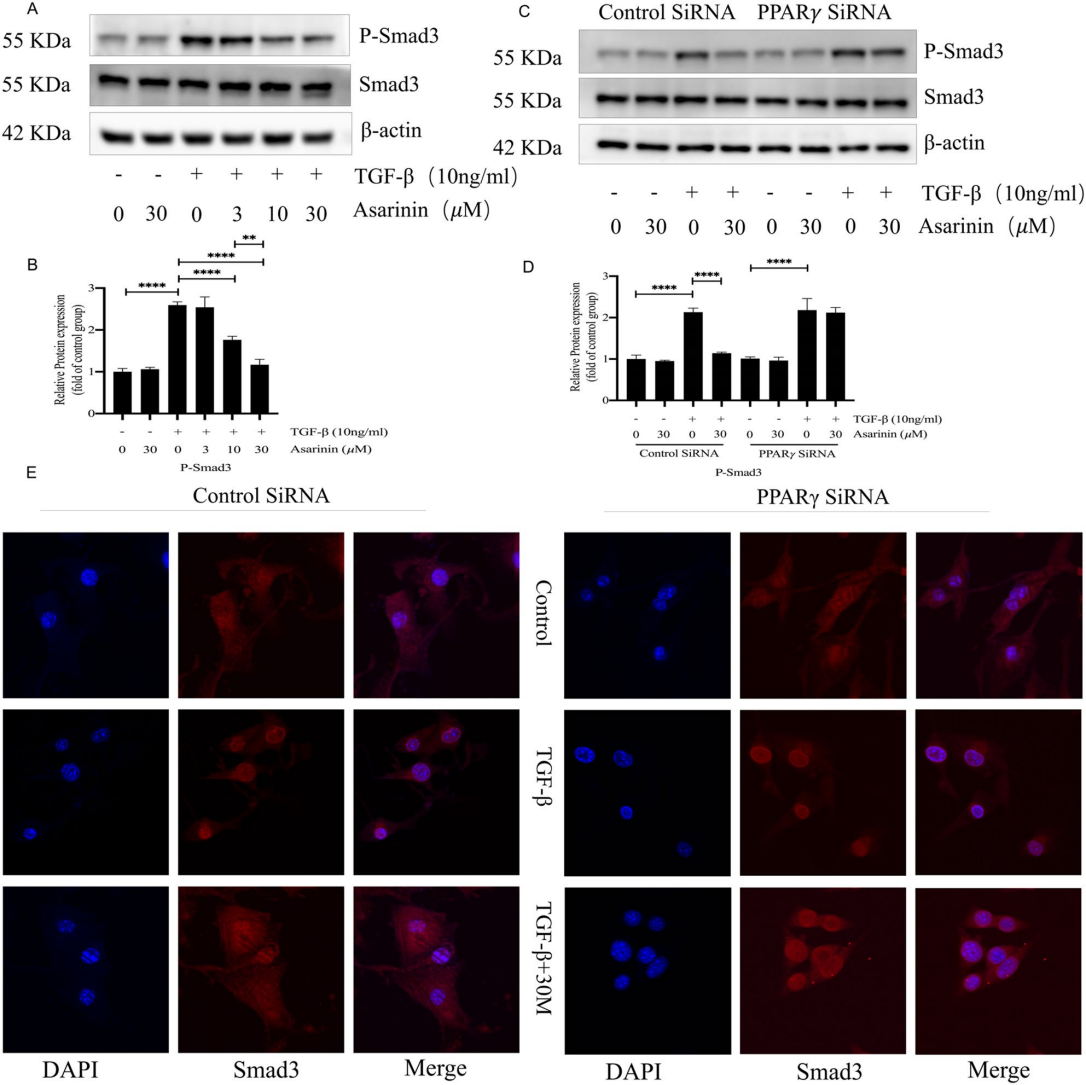
The expression levels of P-Smad3 were reduced by asarinin, and the effect of asarinin on the expression of P-Smad3 was suppressed after PPARγ silencing. Western blotting (WB) detected the protein expression levels of P-Smad3 and Smad3 (**A**,**B**). After silencing the *Pparg*, WB was used to detect the protein expression levels of P-Smad3 and Smad3 (**C**,**D**). Immunofluorescence was used to detect the effect of asarinin on TGF-b-induced Smad3 nuclear translocation (magnification ×1000) (**E**). Data are expressed as mean ± standard deviation, and all experiments were repeated independently at least 3 times, sample size (n) = 3 for each group, *P < 0.05; **P < 0.01; ***P < 0.001; ****P < 0.0001.

PPARγ plays an important role in TGF-β-related Smad pathway. Therefore, we investigated the effect of asarinin on TGF-β-related Smad pathway and found that asarinin effectively reduced the phosphorylation level of Smad3 (Fig. 6A,B). To further investigate whether inhibiting this pathway is related to the activation of PPARγ by asarinin, we specifically silenced *Pparg*. The results showed that the inhibitory effect of asarinin on Smad pathway was suppressed (Fig. 6C,D). To further explore the mechanism of asarinin on the Smad pathway, we found by immunofluorescence that asarinin significantly inhibited TGF-β-induced nuclear translocation of Smad3, and this effect was suppressed after *Pparg* was silenced (Fig. 6E). These results suggest that asarinin inhibited the Smad pathway through PPARγ activation.

### Asarinin inhibited the non-Smad pathway of TGF-β by activating PPARγ

**Figure 7:**
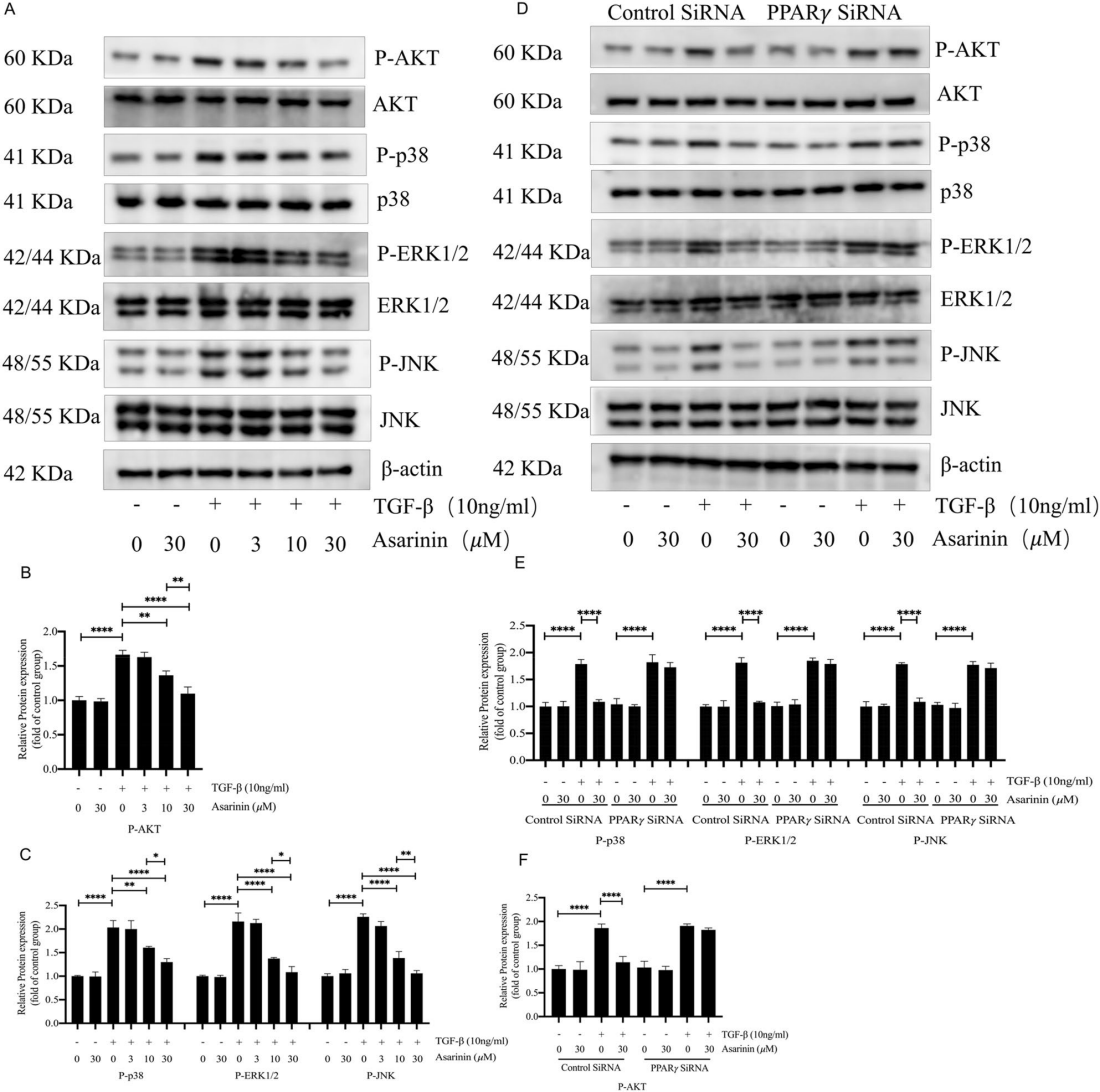
The expression levels of P-AKT, P-p38, P-ERK1/2 and P-JNK were reduced by asarinin, and the effect of asarinin on the expression of P-AKT, P-p38, P-ERK1/2 and P-JNK were suppressed after PPARγ silencing. Western blotting (WB) detected the protein expression levels of P-AKT, AKT, p38, P-p38, ERK1/2, P-ERK1/2, JNK and P-JNK (A-C). After silencing the *Pparg*, WB was used to detect the protein expression levels of P-AKT, AKT, p38, P-p38, ERK1/2, P-ERK1/2, JNK and P-JNK (D-F). Data are expressed as mean ± standard deviation, and all experiments were repeated independently at least 3 times, sample size (n) = 3 for each group, *P < 0.05; **P < 0.01; ***P < 0.001; ****P < 0.0001.

PPARγ plays an important role not only in the TGF-β-related Smad pathway but also in the non-Smad pathways, such as AKT and MAPK pathways^38,39^. Therefore, we also investigated the effects of asarinin on AKT, p38, ERK, and JNK pathways, and found that asarinin effectively reduced their phosphorylation levels (Fig. 7A–C). To further investigate whether inhibiting these pathways is related to the activation of PPARγ by asarinin, we specifically silenced *Pparg*. The results showed that the inhibitory effect of asarinin on these pathways was suppressed (Fig. 7D–F). These results suggest that asarinin inhibited the AKT and MAPK pathways through PPARγ activation.

### PPARγ mediates the antifibrotic effect of asarinin in vivo

**Figure 8:**
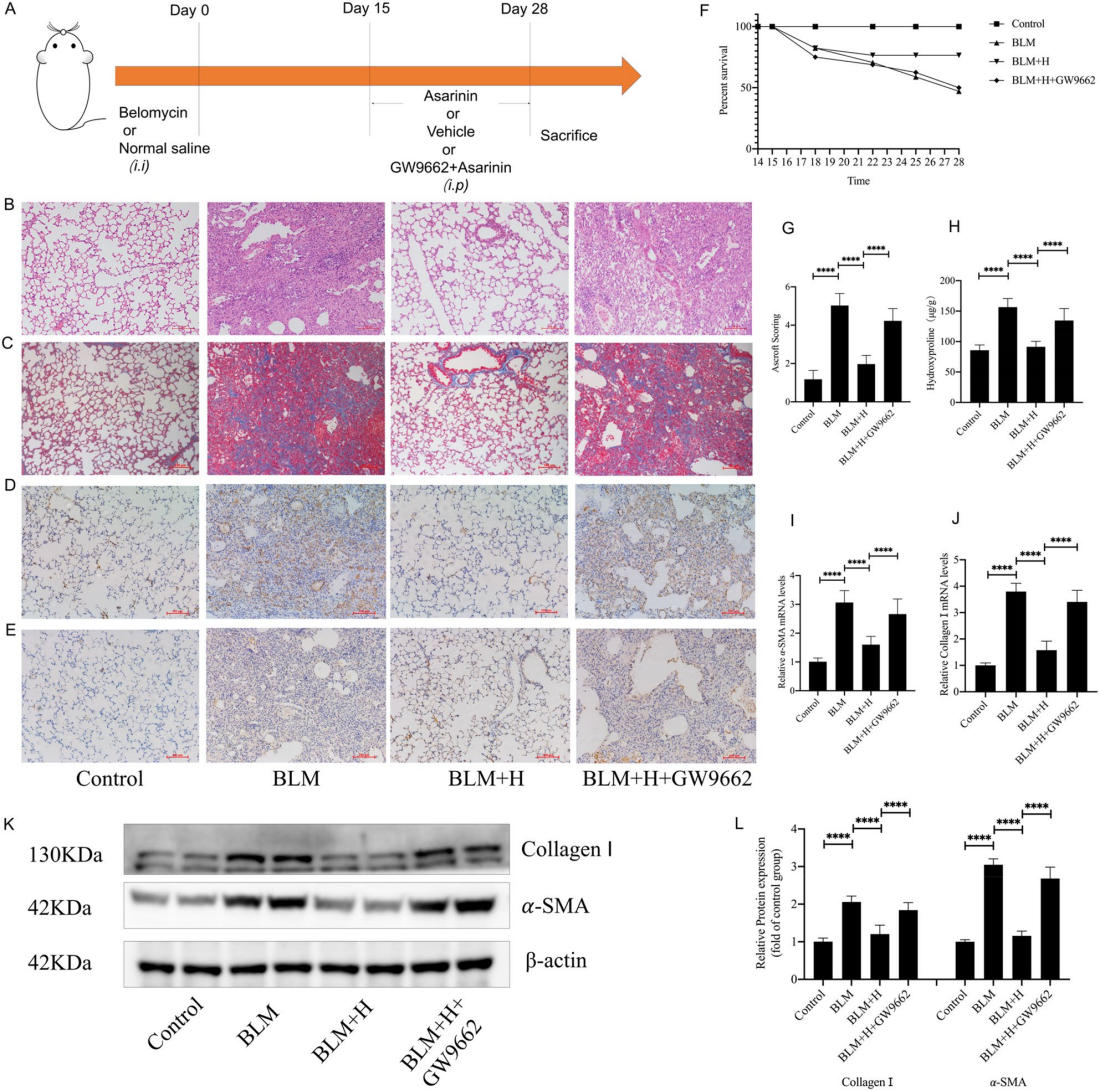
GW9662 inhibited the effect of asarinin on lung tissue morphology and lung fibrosis marker levels. Intraperitoneal injection of 1 mg/kg GW9662 was followed by intraperitoneal injection of 20 mg/kg asarinin 30 min later and compared with pulmonary fibrosis mice and asarinin-treated mice (**A**). Hematoxylin and Eosin staining and Masson staining were used to assess the lung tissue morphology and extracellular matrix deposition (magnification ×100) (**B**,**C**), and the Ashcroft scoring method was used to evaluate the degree of pulmonary fibrosis (**G**). Record the number of deaths in each group of mice and make a survival curve (**F**). Immunohistochemistry was used to assess the expression of α-SMA with type I collagen in lung tissue (magnification ×100) (**D**,**E**). Biochemical methods were used to determine the hydroxyproline content of lung tissue (**H**). Quantitative real-time PCR was used to determine the gene expression levels of *Acta2* and *Col1a1* in lung tissue (**I**,**J**). Western blotting was used to determine the protein expression levels of α-SMA and type I collagen in lung tissue (**K**,**L**). Control represents the control group; BLM represents the pulmonary fibrosis model group; BLM + H represents the high-dose (20 mg/kg) asarinin treatment group; BLM + H + GW9662 represents mice modeled with bleomycin that were injected intraperitoneally with 1 mg/kg of GW9662 prior to each intraperitoneal injection of high-dose asarinin. Data are expressed as mean ± standard deviation, sample size (n) = 8 for each group, *P < 0.05; **P < 0.01; ***P < 0.001; ****P < 0.0001.

Based on the results of our *in vitro* experiments, to further explore whether the inhibitory effect of asarinin on BLM-induced pulmonary fibrosis was related to PPARγ, we pretreated the mice with 1 mg/kg of GW9662 intraperitoneally 30 min before intraperitoneal injection of high-dose asarinin, and then compared the differences between each treatment (Fig. 8A). The results of HE and Masson staining with Ashcroft scoring showed that GW9662 inhibited the effect of asarinin on the morphology and ECM deposition of fibrotic lung tissue (Fig. 8B,C,G). Improvement in survival of pulmonary fibrosis mice by asarinin was also inhibited by GW9662 (Fig. 8F). Similarly, the effect of asarinin on the reduction of hydroxyproline content in lung tissue was also inhibited (Fig. 8H). The results of immunohistochemistry, WB, and Q-PCR showed that GW9662 also inhibited the reduction in α-SMA and type I collagen expression in fibrotic lung tissues (Fig. 8D,E,I–L). These results further demonstrated that PPARγ plays a key role in the antifibrotic effect of asarinin.

## Discussion

Here, we observed for the first time that asarinin inhibited TGF-β1-induced myofibroblast transition by activating PPARγ, thereby attenuating BLM-induced pulmonary fibrosis.

The lung fibrosis model constructed by intratracheal injection of BLM is an important tool for studying the pathological mechanism of IPF and identifying new therapeutic compounds. The number of myofibroblasts and ECM deposition increased after the d 14 of intratracheal injection of BLM in mice, and ECM deposition, including collagen, reached its peak on the d 28^40^. Here, various concentrations of asarinin were injected intraperitoneally over d 15–28 to investigate whether asarinin directly affects anti-pulmonary fibrosis. The results showed that asarinin indeed can directly inhibit pulmonary fibrosis.

Peroxisome proliferator-activated receptors (PPARs) belong to a subfamily of the nuclear receptor superfamily, of which PPARγ is an isoform. PPARs are ligand-dependent transcription factors that regulate the expression of target genes by binding to specific peroxisome response elements at the enhancer sites of controlled genes. Once PPARs are activated, the conformation of PPAR changes and stabilizes, resulting in a binding cleft and recruitment of transcriptional coactivators, resulting in an increase in gene transcription^41,42^. In our study, asarinin activated PPARγ in three kinds of fibroblasts, but whether asarinin acts directly as an activator to bind to the PPARγ receptor has not yet been demonstrated, which also deserves further investigation in subsequent experiments. PPARγ is vital to the development of pulmonary fibrosis, and its activation inhibits the development of various models of pulmonary fibrosis *in vivo*^21–25^. These inhibitory effects involve various mechanisms of action, including the inhibition of fibroblast-to-myofibroblast transition and promoting the lipogenic transformation of myofibroblasts^17,43^. In our study, we demonstrated that asarinin inhibited the transition of various fibroblasts to myofibroblasts through the activation of PPARγ, which may be the mechanism by which it inhibits pulmonary fibrosis. In addition, PPARγ activation can maintain the balance of immune cells, inhibit epithelial cell apoptosis and inhibit epithelial-mesenchymal transition^44,45^, which may also be the mechanisms by which asarinin exerts its antifibrotic effect.

Myofibroblasts play an important role in the development of IPF, as three major factors, including high mechanical stress, increased local activity of TGF-β, and the presence of specific matrix proteins, drive fibroblast differentiation to myofibroblasts, thereby ensuring the maintenance of this phenotype^46^. TGF-β is a major molecular driver of fibrosis and a cytokine that promotes myofibroblast transdifferentiation and ECM production^47,48^. First, TGF-β promotes ECM production and deposition, including directly stimulating myofibroblast formation and the expression of genes associated with ECM, as well as inhibiting matrix metalloproteinase formation for ECM degradation and promoting matrix metalloproteinase inhibitor expression^49,50^. Second, TGF-β promotes alveolar epithelial injury and apoptosis, which crucially influence the development of IPF^51^. The main antifibrotic effect of PPARγ agonists is the inhibition of the TGF-β signaling pathway through PPARγ-dependent effects^52^. Several studies have shown that PPARγ expression affects the TGF-β canonical Smad pathway. Activation of PPARγ decreases the phosphorylation level of Smad2/3 and inhibits the nuclear translocation of Smad2/3, thus inhibiting TGF-β/Smad pathway activation^53,54^. Our study also found that asarinin decreased the phosphorylation level of Smad3 in both *in vivo* and *in vitro* experiments, suggesting that asarinin can exert antifibrotic effects by inhibiting the TGF-β canonical Smad pathway.

In addition to the canonical Smad pathway, TGF-β non-canonical pathways, such as the AKT and MAPK pathways, also play important roles in TGF-β pathway-related biological functions^55^. These pathways play key roles in various biological processes, including inflammatory and fibrotic responses^56–59^. Activation of the AKT pathway is strongly associated with pulmonary fibrosis development in both IPF patients and BLM-induced pulmonary fibrosis models^60–62^. This may be associated with AKT-related endoplasmic reticulum stress, macrophage apoptosis, epithelial cell injury, myofibroblast transition, and ECM formation^63,64^. PPARγ activation also inhibits TGF-β-induced AKT phosphorylation^65^. MAPK and PPARγ pathways are known to interact, and several studies have shown that ERK, JNK, and p38 pathways are activated in BLM models^66–71^; further, increased expression and activation of PPARγ can inhibit these pathways, which may be related to the inhibition of myofibroblast transformation due to PPARγ activation^72–77^. In our study, we also observed in *in vivo* and *in vitro* experiments that the phosphorylation levels of AKT, p38, JNK, and ERK were reduced by asarinin. This suggests that the antifibrotic effect of asarinin is related to its inhibitory effect on the AKT and MAPK pathways.

In our study, we found that asarinin activated PPARγ and inhibited canonical and non-canonical TGF-β pathways. This may be due to the activation of PPARγ by asarinin, or it may be due to the direct inhibition of TGF-β receptor activity by asarinin, which leads to the inhibition of the canonical and non-canonical pathways. In the *in vitro* experiments, *Pparg* silencing inhibited the effects of asarinin on Smad, AKT and MAPK pathways. Therefore, we speculated that PPARγ is a key target by which asarinin exerts its antifibrotic effect, which is related not only to the inhibitory effect of PPARγ on the TGF-β canonical pathway, but also to its inhibitory effect on AKT and MAPK pathways in non-canonical pathways.

Sesamin is a stereoisomer of asarinin, and they have a similar spatial structure and similar biological activities^78^. Multiple studies have shown that sesamin can effectively inhibit the progression of fibrosis in various fibrosis animal models^79–81^. In our study, the anti-pulmonary fibrosis effect of asarinin was demonstrated. These results all provide strong evidence for the application of asarinin in clinical treatment of pulmonary fibrosis in the future.

## Conclusions

By observing the activation of PPARγ by asarinin, we elucidated the mechanism by which it exerts its antifibrotic effect. By activating PPARγ, asarinin inhibits the TGF-β canonical Smad pathway and the non-canonical AKT and MAPK pathways, thereby inhibiting the transition of fibroblasts to myofibroblasts, and thus lung fibrosis. Our study shows for the first time that asarinin exerts antifibrotic effects by activating PPARγ, providing evidence that asarinin may be useful for the treatment of clinical pulmonary fibrosis.

### Supplementary Information

Supplementary Information.

## Data Availability

All co-authors of this study agreed to disclose all data from the study and all data generated or analyzed in the course of this study are included in this manuscript and its supplements. Any supporting data the article is available from the corresponding author upon reasonable request.

## References

[1] Lederer DJ, Martinez FJ (2018). Idiopathic pulmonary fibrosis. N. Engl. J. Med..

[2] Raghu G (2022). Idiopathic pulmonary fibrosis (an update) and progressive pulmonary fibrosis in adults: An official ATS/ERS/JRS/ALAT clinical practice guideline. Am. J. Respir. Crit. Care Med..

[3] Albert RK, Schwartz DA (2019). Revealing the secrets of idiopathic pulmonary fibrosis. N. Engl. J. Med..

[4] Tomasek JJ, Gabbiani G, Hinz B, Chaponnier C, Brown RA (2002). Myofibroblasts and mechano-regulation of connective tissue remodelling. Nat. Rev. Mol. Cell Biol..

[5] Marchioni A (2021). Pulmonary stretch and lung mechanotransduction: Implications for progression in the fibrotic lung. Int. J. Mol. Sci..

[6] Hinz B (2016). Myofibroblasts. Exp. Eye Res..

[7] Border WA, Noble NA (1994). Transforming growth factor beta in tissue fibrosis. N. Engl. J. Med..

[8] Blobe GC, Schiemann WP, Lodish HF (2000). Role of transforming growth factor beta in human disease. N. Engl. J. Med..

[9] Aschner Y, Downey GP (2016). Transforming growth factor-beta: Master regulator of the respiratory system in health and disease. Am. J. Respir. Cell Mol. Biol..

[10] Wolters PJ, Collard HR, Jones KD (2014). Pathogenesis of idiopathic pulmonary fibrosis. Annu. Rev. Pathol..

[11] Zhang XL, Xing RG, Chen L, Liu CR, Miao ZG (2016). PI3K/Akt signaling is involved in the pathogenesis of bleomycin-induced pulmonary fibrosis via regulation of epithelial-mesenchymal transition. Mol. Med. Rep..

[12] Ye Z, Hu Y (2021). TGF-beta1: Gentlemanly orchestrator in idiopathic pulmonary fibrosis (Review). Int. J. Mol. Med..

[13] Yang JY (2019). Wedelolactone attenuates pulmonary fibrosis partly through activating AMPK and regulating Raf-MAPKs signaling pathway. Front. Pharmacol..

[14] Hsu HS (2017). Involvement of ER stress, PI3K/AKT activation, and lung fibroblast proliferation in bleomycin-induced pulmonary fibrosis. Sci. Rep..

[15] Berger J, Moller DE (2002). The mechanisms of action of PPARs. Annu. Rev. Med..

[16] Westin S (1998). Interactions controlling the assembly of nuclear-receptor heterodimers and co-activators. Nature.

[17] Hua Q (2021). PPARgamma mediates the anti-pulmonary fibrosis effect of icaritin. Toxicol. Lett..

[18] Ghosh AK (2004). Disruption of transforming growth factor beta signaling and profibrotic responses in normal skin fibroblasts by peroxisome proliferator-activated receptor gamma. Arthritis Rheum..

[19] Kokeny G, Calvier L, Hansmann G (2021). PPARgamma and TGFbeta-major regulators of metabolism, inflammation, and fibrosis in the lungs and kidneys. Int. J. Mol. Sci..

[20] Wang Y (2000). A synthetic triterpenoid, 2-cyano-3,12-dioxooleana-1,9-dien-28-oic acid (CDDO), is a ligand for the peroxisome proliferator-activated receptor gamma. Mol. Endocrinol..

[21] Li A (2016). Activating peroxisome proliferator-activated receptors (PPARs): A new sight for chrysophanol to treat paraquat-induced lung injury. Inflammation.

[22] Routh R, Johnson J, McCarthy K (2002). Troglitazone suppresses the secretion of type I collagen by mesangial cells in vitr. Kidney Int..

[23] Aoki Y (2009). Pioglitazone, a peroxisome proliferator-activated receptor gamma ligand, suppresses bleomycin-induced acute lung injury and fibrosis. Respiration.

[24] Cui MX, Chen XL, Chen C, Hu XJ, Jin H (2010). Effects of rosiglitazone on the expression of connective tissue growth factor in the pulmonary arteries of rats suffering from fibrosis in lung. Zhongguo Ying Yong Sheng Li Xue Za Zhi.

[25] Rangarajan S (2018). Metformin reverses established lung fibrosis in a bleomycin model. Nat. Med..

[26] Li J, Guo C, Wu J (2021). The agonists of peroxisome proliferator-activated receptor-gamma for liver fibrosis. Drug Des. Dev. Ther..

[27] Liu HJ, Liao HH, Yang Z, Tang QZ (2016). Peroxisome proliferator-activated receptor-gamma is critical to cardiac fibrosis. PPAR Res..

[28] Dantas AT (2015). The role of PPAR gamma in systemic sclerosis. PPAR Res..

[29] Jing Y (2017). Chemical constituents from the roots and rhizomes of Asarum heterotropoides var. mandshuricum and the in vitro anti-inflammatory activity. Molecules.

[30] Park HJ, Lee KS, Zhao TT, Lee KE, Lee MK (2017). Effects of asarinin on dopamine biosynthesis and 6-hydroxydopamine-induced cytotoxicity in PC12 cells. Arch. Pharm. Res..

[31] Dai Q, Wang M, Li Y, Li J (2019). Amelioration of CIA by asarinin is associated to a downregulation of TLR9/NF-kappaB and regulation of Th1/Th2/Treg expression. Biol. Pharm. Bull..

[32] Gu J (2015). The effect of Asarinin on Toll-like pathway in rats after cardiac allograft implantation. Transplant. Proc..

[33] Hou Y (2020). (-)-Asarinin inhibits mast cells activation as a Src family kinase inhibitor. Int. J. Biochem. Cell Biol..

[34] Jeong M, Kim HM, Lee JS, Choi JH, Jang DS (2018). (-)-Asarinin from the roots of asarum sieboldii induces apoptotic cell death via caspase activation in human ovarian cancer cells. Molecules.

[35] Ashcroft T, Simpson JM, Timbrell V (1988). Simple method of estimating severity of pulmonary fibrosis on a numerical scale. J. Clin. Pathol..

[36] Moeller A, Ask K, Warburton D, Gauldie J, Kolb M (2008). The bleomycin animal model: A useful tool to investigate treatment options for idiopathic pulmonary fibrosis?. Int. J. Biochem. Cell Biol..

[37] Liu T, De Los SF, Phan SH (2017). The bleomycin model of pulmonary fibrosis. Methods Mol. Biol..

[38] Zhu W (2016). PPAR-gamma agonist pioglitazone regulates dendritic cells immunogenicity mediated by DC-SIGN via the MAPK and NF-kappaB pathways. Int. Immunopharmacol..

[39] Ma ZG (2017). Piperine attenuates pathological cardiac fibrosis via PPAR-gamma/AKT pathways. EBioMedicine.

[40] Kolb P (2020). The importance of interventional timing in the bleomycin model of pulmonary fibrosis. Eur. Respir. J.

[41] Janani C, Ranjitha KB (2015). PPAR gamma gene–a review. Diabetes Metab. Syndr..

[42] Carvalho MV, Goncalves-de-Albuquerque CF, Silva AR (2021). PPAR gamma: From definition to molecular targets and therapy of lung diseases. Int. J. Mol. Sci..

[43] Kheirollahi V (2019). Metformin induces lipogenic differentiation in myofibroblasts to reverse lung fibrosis. Nat. Commun..

[44] Lin Y, Yang P (2021). Phillygenin inhibits the inflammation and apoptosis of pulmonary epithelial cells by activating PPARgamma signaling via downregulation of MMP8. Mol. Med. Rep..

[45] Ivanova EA (2015). Peroxisome proliferator-activated receptor (PPAR) gamma in cardiovascular disorders and cardiovascular surgery. J. Cardiol..

[46] Hinz B (2007). The myofibroblast: One function, multiple origins. Am. J. Pathol..

[47] Leask A, Abraham DJ (2004). TGF-beta signaling and the fibrotic response. FASEB J..

[48] Willis BC, Borok Z (2007). TGF-beta-induced EMT: Mechanisms and implications for fibrotic lung disease. Am. J. Physiol. Lung Cell. Mol. Physiol..

[49] Park SA (2015). EW-7197 inhibits hepatic, renal, and pulmonary fibrosis by blocking TGF-beta/Smad and ROS signaling. Cell. Mol. Life Sci..

[50] Clarke DL, Carruthers AM, Mustelin T, Murray LA (2013). Matrix regulation of idiopathic pulmonary fibrosis: The role of enzymes. Fibrogenesis Tissue Repair.

[51] Jarman ER (2014). An inhibitor of NADPH oxidase-4 attenuates established pulmonary fibrosis in a rodent disease model. Am. J. Respir. Cell Mol. Biol..

[52] Deng YL, Xiong XZ, Cheng NS (2012). Organ fibrosis inhibited by blocking transforming growth factor-beta signaling via peroxisome proliferator-activated receptor gamma agonists. Hepatobiliary Pancreat. Dis. Int..

[53] Wu X (2022). Effectiveness and mechanism of metformin in animal models of pulmonary fibrosis: A preclinical systematic review and meta-analysis. Front. Pharmacol..

[54] Calvier L (2017). PPARgamma links BMP2 and TGFbeta1 pathways in vascular smooth muscle cells, regulating cell proliferation and glucose metabolism. Cell Metab..

[55] Ji Y (2016). Paeoniflorin suppresses TGF-beta mediated epithelial-mesenchymal transition in pulmonary fibrosis through a Smad-dependent pathway. Acta Pharmacol. Sin..

[56] Cargnello M, Roux PP (2011). Activation and function of the MAPKs and their substrates, the MAPK-activated protein kinases. Microbiol. Mol. Biol. Rev..

[57] Yoshida K (2002). MAP kinase activation and apoptosis in lung tissues from patients with idiopathic pulmonary fibrosis. J. Pathol..

[58] Li X, Ye C, Mulati M, Sun L, Qian F (2020). Ellipticine blocks synergistic effects of IL-17A and TNF-alpha in epithelial cells and alleviates severe acute pancreatitis-associated acute lung injury. Biochem. Pharmacol..

[59] Nie Y (2019). S-allyl-l-cysteine attenuates bleomycin-induced pulmonary fibrosis and inflammation via AKT/NF-kappaB signaling pathway in mice. J. Pharmacol. Sci..

[60] Wan H (2019). Thy-1 depletion and integrin beta3 upregulation-mediated PI3K-Akt-mTOR pathway activation inhibits lung fibroblast autophagy in lipopolysaccharide-induced pulmonary fibrosis. Lab. Invest..

[61] Hu X (2020). PI3K-Akt-mTOR/PFKFB3 pathway mediated lung fibroblast aerobic glycolysis and collagen synthesis in lipopolysaccharide-induced pulmonary fibrosis. Lab. Invest..

[62] Larson-Casey JL, Deshane JS, Ryan AJ, Thannickal VJ, Carter AB (2016). Macrophage Akt1 kinase-mediated mitophagy modulates apoptosis resistance and pulmonary fibrosis. Immunity.

[63] Wang J (2022). Targeting PI3K/AKT signaling for treatment of idiopathic pulmonary fibrosis. Acta Pharm. Sin. B.

[64] Hohmann MS, Habiel DM, Coelho AL, Verri WJ, Hogaboam CM (2019). Quercetin enhances ligand-induced apoptosis in senescent idiopathic pulmonary fibrosis fibroblasts and reduces lung fibrosis in vivo. Am. J. Respir. Cell Mol. Biol..

[65] Kulkarni AA (2011). PPAR-gamma ligands repress TGFbeta-induced myofibroblast differentiation by targeting the PI3K/Akt pathway: Implications for therapy of fibrosis. PLOS ONE.

[66] Huang X (2016). Baicalin attenuates bleomycin-induced pulmonary fibrosis via adenosine A2a receptor related TGF-beta1-induced ERK1/2 signaling pathway. BMC Pulm. Med..

[67] Zhang YP (2012). siRNA against plasminogen activator inhibitor-1 ameliorates bleomycin-induced lung fibrosis in rats. Acta Pharmacol. Sin..

[68] Xiong Y (2021). Dehydrocostus lactone inhibits BLM-induced pulmonary fibrosis and inflammation in mice via the JNK and p38 MAPK-mediated NF-kappaB signaling pathways. Int. Immunopharmacol..

[69] Tong X (2021). Azithromycin attenuates bleomycin-induced pulmonary fibrosis partly by inhibiting the expression of LOX and LOXL-2. Front. Pharmacol..

[70] Zhu L (2021). The antioxidant N-acetylcysteine promotes immune response and inhibits epithelial-mesenchymal transition to alleviate pulmonary fibrosis in chronic obstructive pulmonary disease by suppressing the VWF/p38 MAPK axis. Mol. Med..

[71] Valenca SS, Dong BE, Gordon EM, Sun RC, Waters CM (2022). ASK1 regulates bleomycin-induced pulmonary fibrosis. Am. J. Respir. Cell Mol. Biol..

[72] Zhang X (2022). Different dose of sucrose consumption divergently influences gut microbiota and PPAR-gamma/MAPK/NF-kappaB pathway in DSS-induced colitis mice. Nutrients.

[73] Zhu W (2021). Vitamin D3 alleviates pulmonary fibrosis by regulating the MAPK pathway via targeting PSAT1 expression in vivo and in vitro. INT Immunopharmacol..

[74] Wang J (2018). Resveratrol inhibits pulmonary fibrosis by regulating miR-21 through MAPK/AP-1 pathways. Biomed. Pharmacother..

[75] Liu T, Gonzalez DLSF, Hirsch M, Wu Z, Phan SH (2021). Noncanonical Wnt signaling promotes myofibroblast differentiation in pulmonary fibrosis. Am. J. Respir. Cell Mol. Biol..

[76] Axmann A (1998). Transforming growth factor-beta1-induced activation of the Raf-MEK-MAPK signaling pathway in rat lung fibroblasts via a PKC-dependent mechanism. Biochem. Biophys. Res. Commun..

[77] Finlay GA, Thannickal VJ, Fanburg BL, Paulson KE (2000). Transforming growth factor-beta 1-induced activation of the ERK pathway/activator protein-1 in human lung fibroblasts requires the autocrine induction of basic fibroblast growth factor. J. Biol. Chem..

[78] Li CY, Chow TJ, Wu TS (2005). The epimerization of sesamin and asarinin. J. Nat. Prod..

[79] Chen X, Ying X, Chen L, Zhang W, Zhang Y (2015). Protective effects of sesamin on liver fibrosis through antioxidative and anti-inflammatory activities in rats. Immunopharmacol. Immunotoxicol..

[80] Zhao M, Zheng S, Yang J (2015). Suppression of TGF-beta1/Smad signaling pathway by sesamin contributes to the attenuation of myocardial fibrosis in spontaneously hypertensive rats. PLOS ONE.

[81] Fan D (2017). Sesamin protects against cardiac remodeling via Sirt3/ROS pathway. Cell. Physiol. Biochem..

