# Supplementary material for: Asarinin attenuates bleomycin-induced pulmonary fibrosis by activating PPARγ
Source: Sci Rep. 2023 Sep 7;13:14706. doi: 10.1038/s41598-023-41933-5 (PMC10485066; doi:10.1038/s41598-023-41933-5)
Supplement: Supplementary file 1 — Supplementary Information. [file 41598_2023_41933_MOESM1_ESM.pdf]

1. Asarinin attenuated BLM-induced pulmonary fibrosis in mice

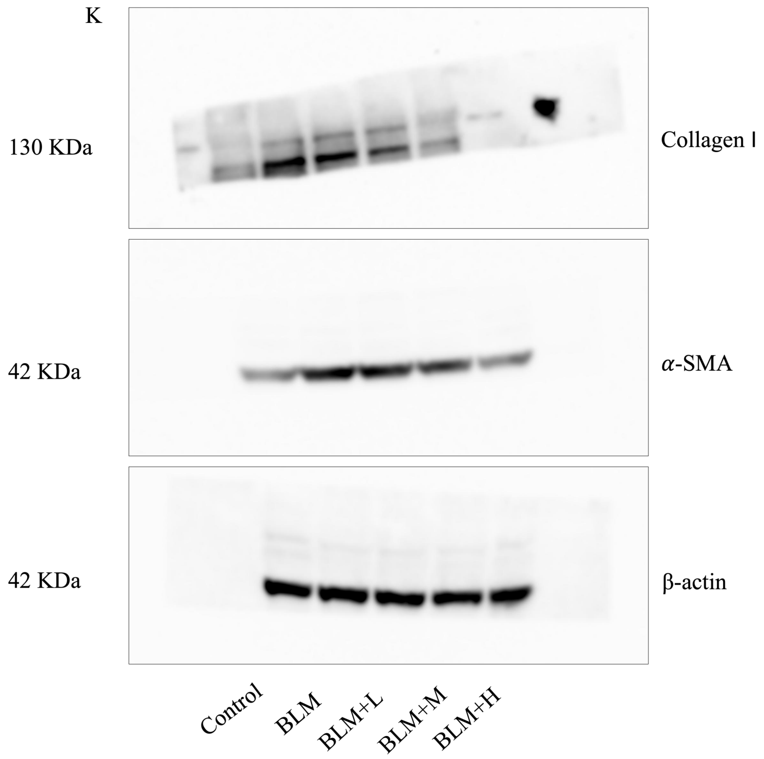

Figure 1. Asarinin improved lung tissue morphology and structure and reduced lung fibrosis marker levels. Western blot analysis of α-SMA and type I collagen protein levels in lung tissue (K).

2. Asarinin attenuated the reduction of PPARγ and activation of Smad, AKT, and MAPK in BLM-induced pulmonary fibrosis in mice

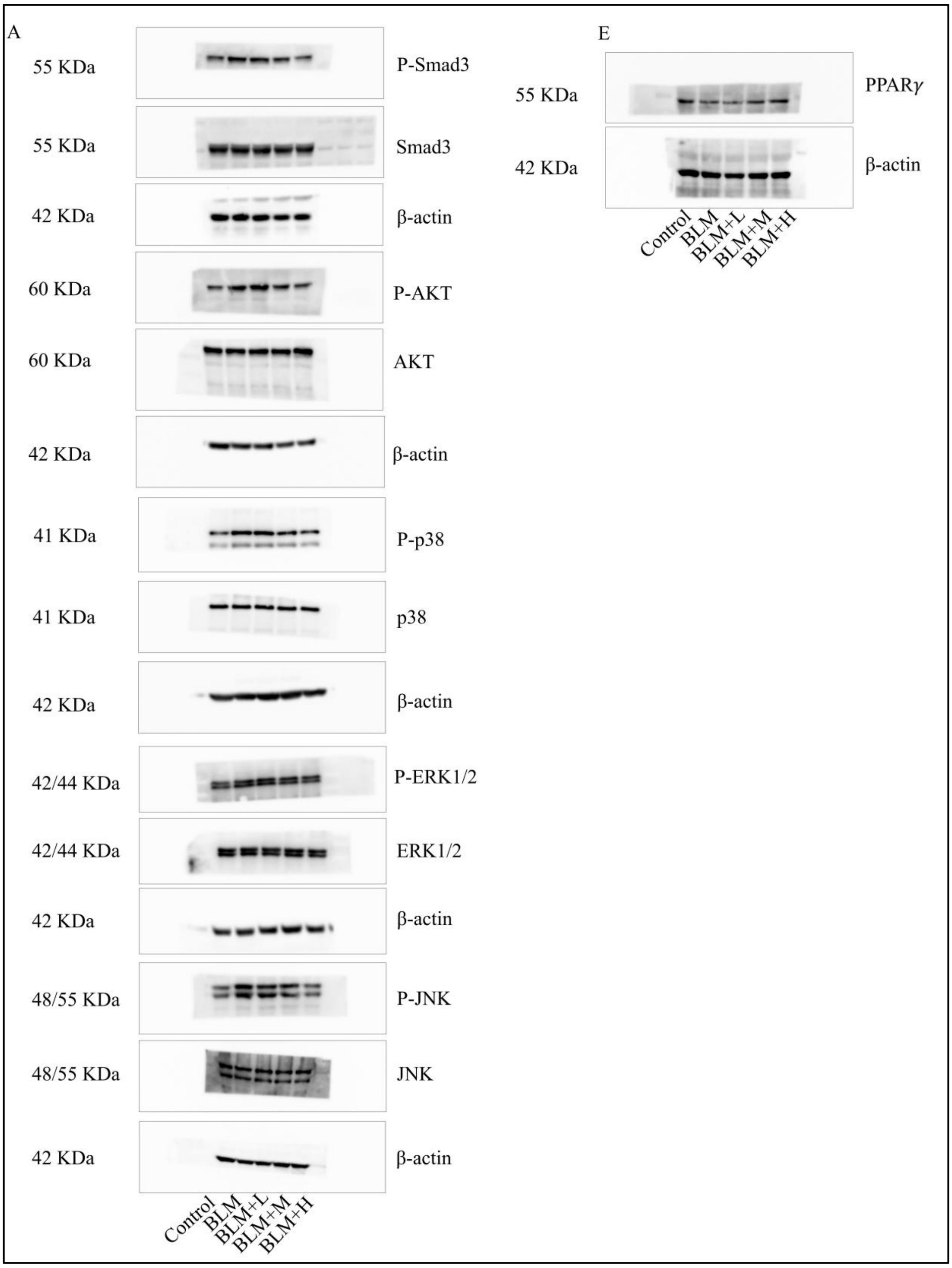

Figure 2. Asarinin attenuated the decrease in expression of PPARγ and increase in phosphorylation levels of Smad3, AKT, p38, ERK1/2 and JNK caused by BLM. Western blotting analysis for protein expression levels of P-Smad3, Smad3, P-AKT, AKT, p38, P-p38, ERK1/2, P-ERK1/2, JNK, P-JNK and PPARγ protein expression levels in mouse lung tissues (A, E).

3. Asarinin inhibited TGF-β1-induced fibroblast-to-myofibroblast transition

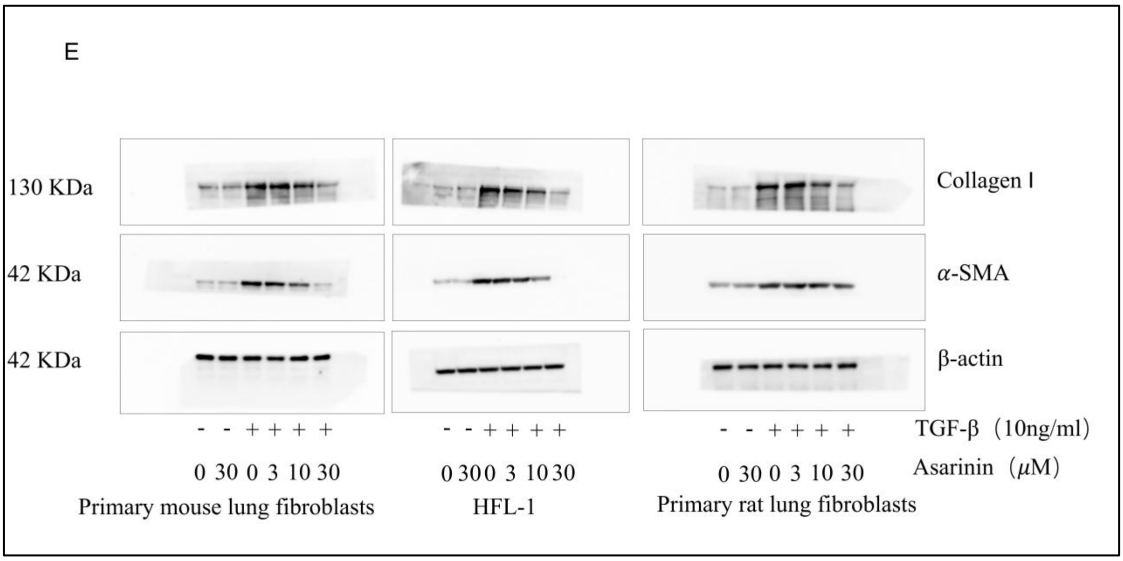

Figure 3. Asarinin decreased the expression levels of TGF-β1-induced α-SMA and type I collagen. Western blotting analysis for the protein expression levels of α-SMA and type I collagen in the three types of cells induced by TGF-β1 (E).

4. Asarinin promoted PPARγ expression and activated PPARγ

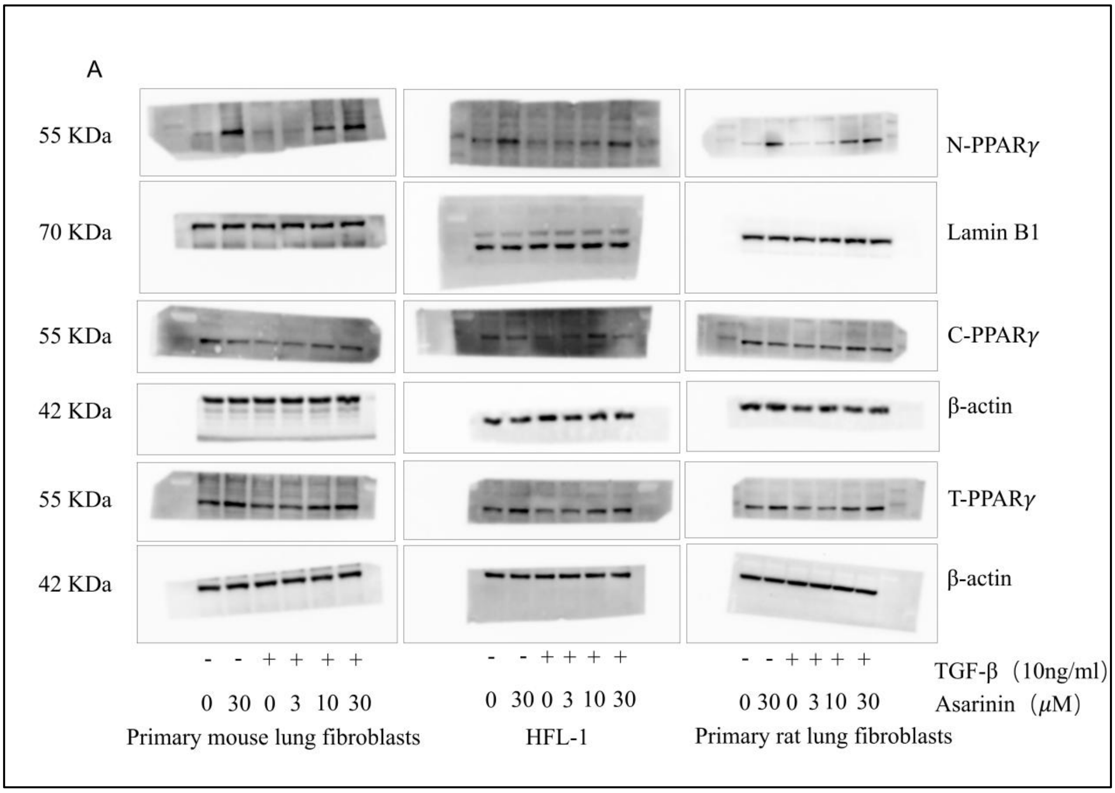

Figure 4. Asarinin promoted the expression and nuclear translocation of PPARγ. After extracting total cellular, nuclear, and cytoplasmic proteins, we verified their PPARγ protein expression levels by western blotting (A).

5. PPARγ mediates the antifibrotic effect of asarinin

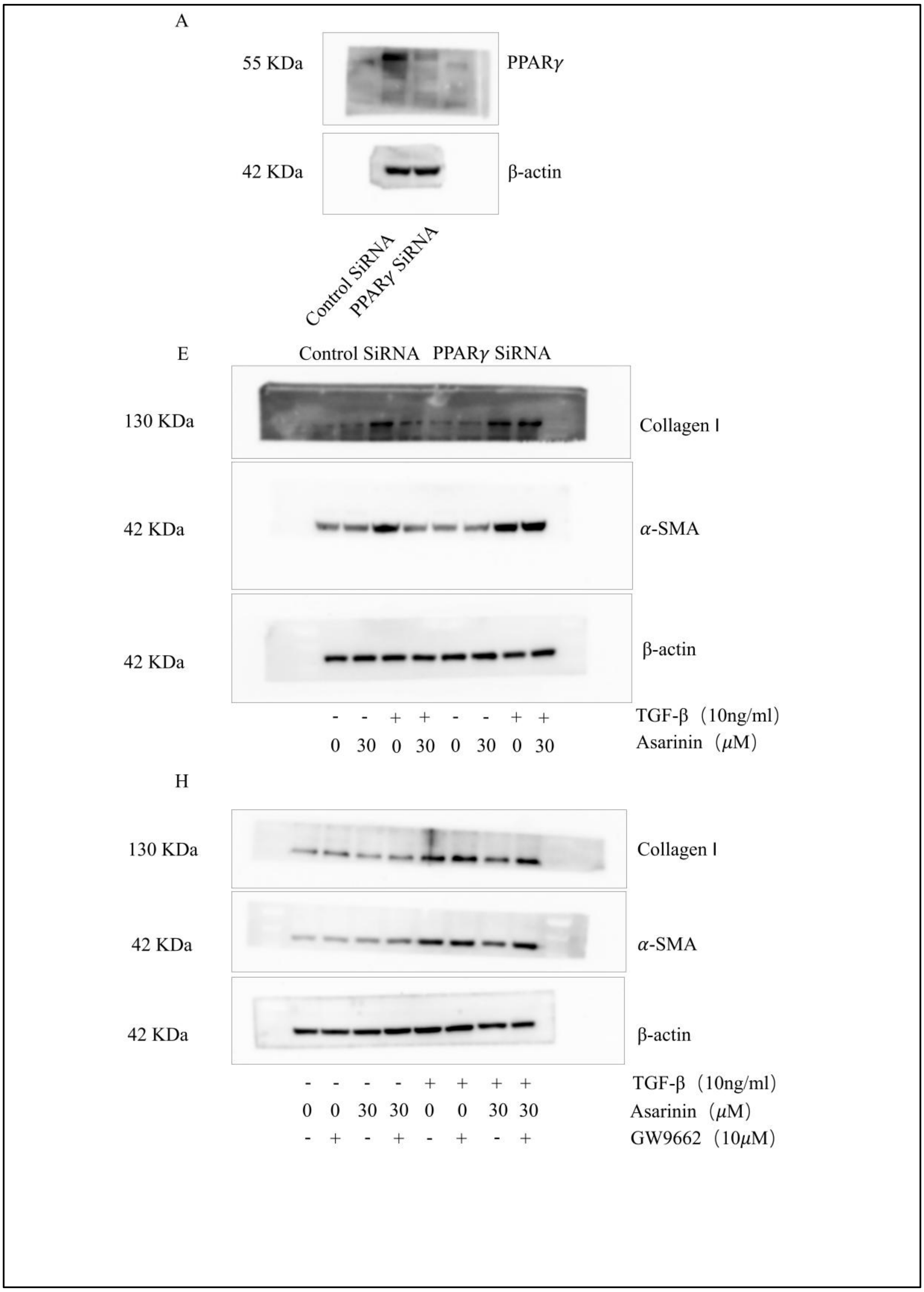

Figure 5. GW9662 and Pparg silencing inhibited the effect of asarinin on α-SMA and type I collagen expression in myofibroblast transition. Western blotting (WB) verified the effect of Pparg silencing (A). WB detected the protein expression levels of α-SMA and type I collagen after Pparg silencing or GW9662 co-incubation (E, H).

6. Asarinin inhibited the Smad pathway of TGF-β by activating PPARγ

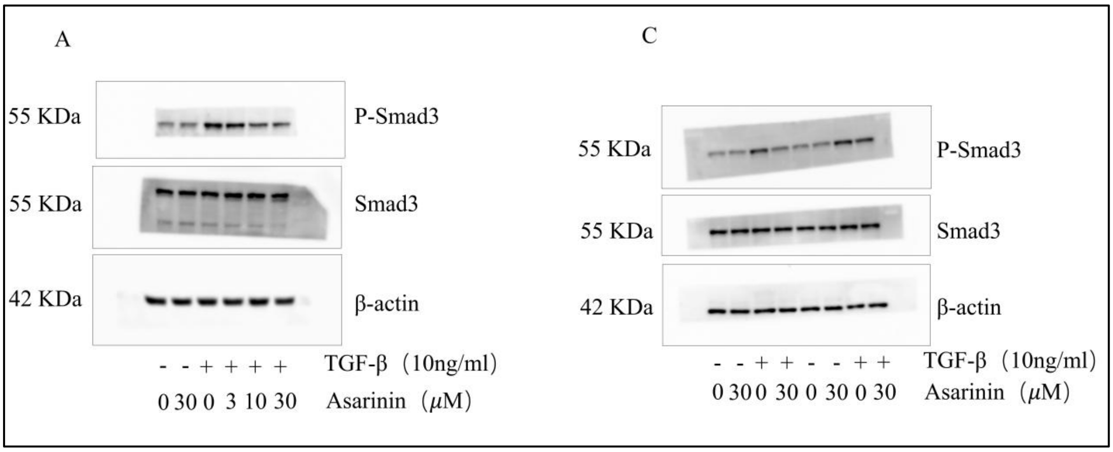

Figure 6. The expression levels of P-Smad3 were reduced by asarinin, and the effect of asarinin on the expression of P-Smad3 was suppressed after PPARγ silencing. Western blotting (WB) detected the protein expression levels of P-Smad3 and Smad3 (A). After silencing the Pparg, WB was used to detect the protein expression levels of P-Smad3 and Smad3 (C).

7. Asarinin inhibited the non-Smad pathway of TGF-β by activating PPARγ

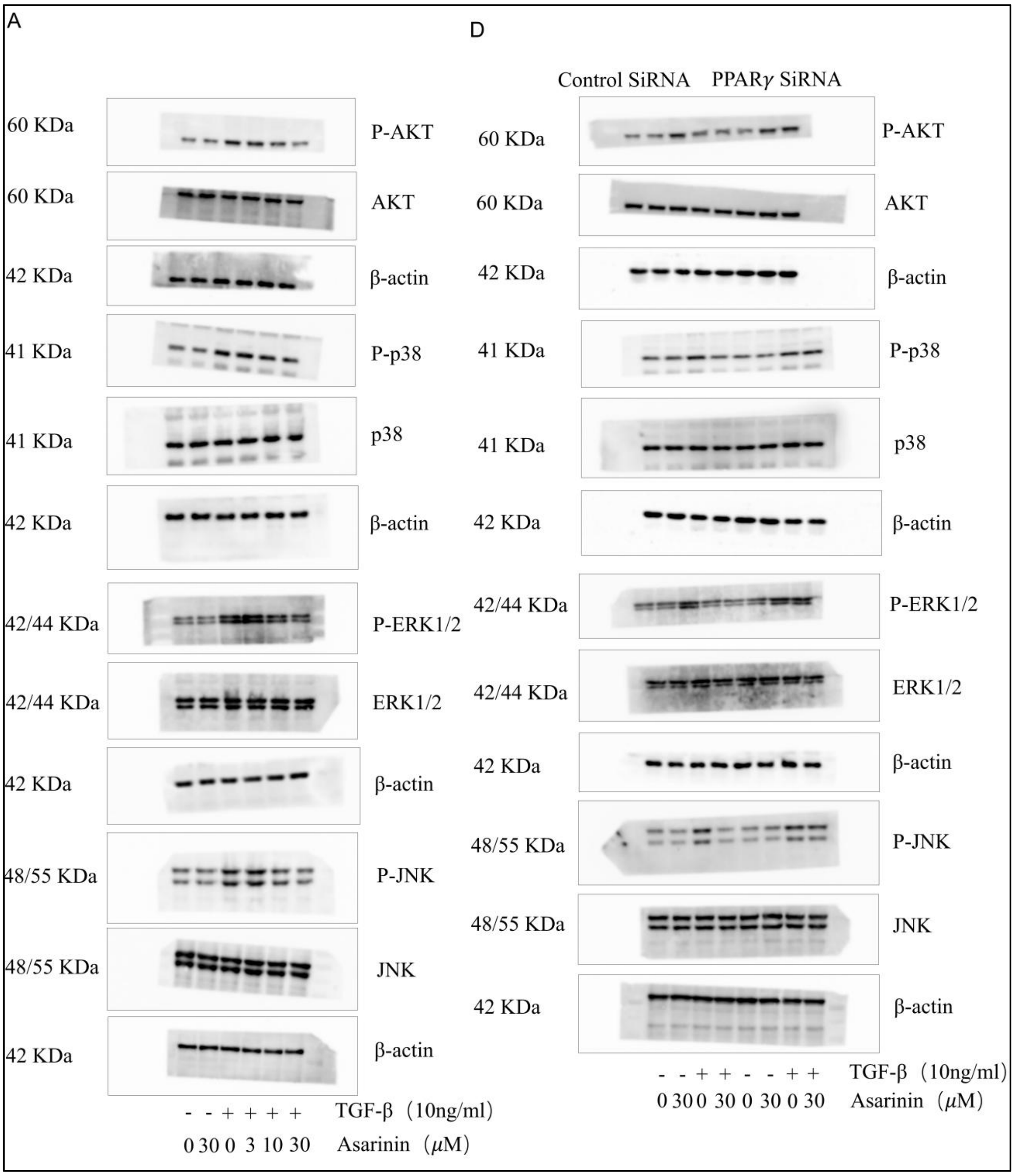

Figure 7. The expression levels of P-AKT, P-p38, P-ERK1/2 and P-JNK were reduced by asarinin, and the effect of asarinin on the expression of P-AKT, P-p38, P-ERK1/2 and P-JNK were suppressed after PPARγ silencing. Western blotting (WB) detected the protein expression levels of P-AKT, AKT, p38, P-p38, ERK1/2, P-ERK1/2, JNK and P-JNK (A). After silencing the Pparg, WB was used to detect the protein ex-pression levels of P-AKT, AKT, p38, P-p38, ERK1/2, P-ERK1/2, JNK and P-JNK (D).

8. PPARγ mediates the antifibrotic effect of asarinin in vivo

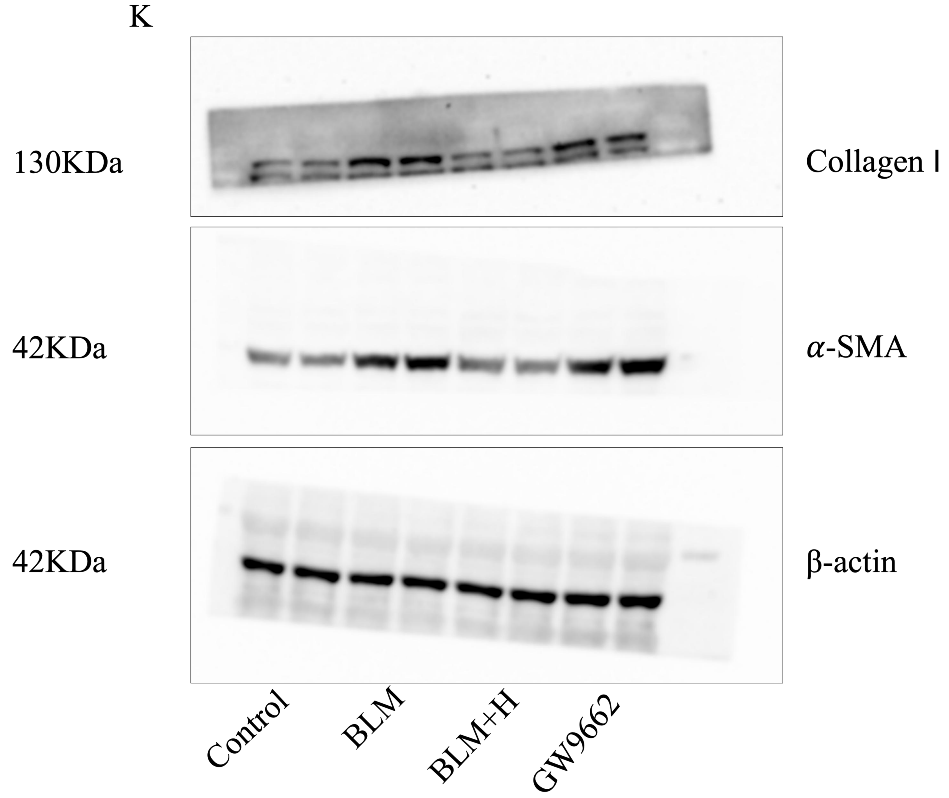

Figure 8. GW9662 inhibited the effect of asarinin on lung tissue morphology and lung fibrosis marker levels. Western blotting was used to determine the protein expression levels of α-SMA and type I colla-gen in lung tissue (K).
